# Der Skorbut – eine heute vergessene Volkskrankheit

**DOI:** 10.1007/s10354-024-01054-8

**Published:** 2024-10-16

**Authors:** Heinz Flamm

**Affiliations:** Medizinische Universität Wien, Martinstraße 7, 3400 Klosterneuburg, Österreich

**Keywords:** Seeskorbut, Landskorbut, Endemien in „feuchten“ Gegenden, Skorbut beim k. k. Militär, Morbus Möller-Barlow, Sea scurvy, Land scurvy, Endemics in “wet” countries, Scurvy in the Austrian Army, Möller-Barlow disease

## Abstract

Als im 15. Jahrhundert neben den bisherigen küstennahen Seefahrten mit leicht erreichbarer frischer Kost die oft jahrelangen Seereisen nur mit konservierten Lebensmitteln (Fische, Salzfleisch, Zwieback) unternommen wurden, trat nach wenigen Wochen bei den Besatzungen eine oft tödliche Krankheit mit Hinfälligkeit, Ausfall der Zähne und fürchterlichem Gestank aus dem Mund auf.

Bald mehrten sich Berichte über gleiche Krankheitsfälle in den Niederlanden, Norddeutschland und im Baltikum, in Frankreich, Lothringen und Genf, in Böhmen, Mähren, Schlesien, Schwaben und Russland: der endemische „Landskorbut“. Als erste ärztliche Beschreibung gilt ein 1541 von Johann Echt an Kollegen im niederdeutschen Raum geschriebener Brief, der viermal publiziert worden ist. Eine der ersten umfänglichen Bearbeitungen lieferte der englische Arzt James Lind. Über den Skorbut, wurde unter etlichen lokalen Namen, wie Scharbock, Scheurbuik oder Scurvy berichtet.

Unter den pathologischen Veränderungen dominierten die Nekrosen der Gingiva mit Zahnausfall, die Blutungen mit Zerstörungen an den Knorpel-Knochen-Grenzen insbesondere der Rippen und die subperiostalen Blutungen der langen Knochen und Rippen.

Zur Behandlung und Vorbeugung des Skorbuts bewährte sich bald der Konsum von grünen Pflanzen und frischen tierischen Eingeweiden. Die lange Zeit bevorzugten Pflanzen waren Scharbockskraut, Großes Schöllkraut und Löffelkraut. Aber auch andere grüne Pflanzen und der Absud von Koniferen-Nadeln wirkten. Nachdem der primär in England als „akute Rachitis“ beschriebene Morbus Möller-Barlow als kindlicher Skorbut erkannt war, befasste man sich auch mit der Zubereitung der Milch zur Vermeidung der Krankheit. Schließlich fand man die Hexuronsäure, die bald unter dem Namen Askorbinsäure oder Vitamin C als gut dosierbares Mittel zur Bekämpfung des Skorbuts allgemein verfügbar wurde.

## Abstract (en)

From the sea voyages of the fifteenth century often lasting for years with provisions only of fish, salted meat and biscuits reports came of fatal health alterations of the seamen which were characterized by prostration, loss of teeth and bad breath.

Reports on comparable endemic illnesses soon came from the Netherlands, North Germany, the Baltic area, France, Lorraine, Geneva, Bohemia, Moravia, Silesia, Swabia and Russia: the so-called land scurvy. The first medical specification was found in a letter from 1541 by Johann Echt to colleges in the area of Lower Germany, which was published four times. One of the first comprehensive considerations was prepared by the English physician James Lind. Scurvy was also mostly published under many local names, such as Skorbut, Scharbock or Scheurbuik.

The pathology of scurvy is dominated by gingival necrosis with loss of teeth, hemorrhages with destruction of the cartilage-bone border especially of the ribs and by subperiosteal hemorrhages of the ribs and long bones.

For treatment and prevention of scurvy eating green plants as originally *Ficaria verna* (lesser celandine, pilewort), *Chelidonium majus* (great celandine, nipplewort, tetterwort) and *Cochlearia officinalis* (scurvy grass) proved to be effective. Where green plants were missing decoctions of conifer needles and fresh animal innards were used. When the infantile disease originally named “acute rachitis” in England was diagnosed as scurvy (later Möller-Barlow disease) the treatment of cow′s milk for baby food became of great interest.

Finally, hexuronic acid, later called ascorbic acid or vitamin C, was found to be a drug that could easily dosed for curing and prevention of scurvy.

Nach den Abhandlungen von Pellagra und Ergotismus (Flamm [1, 2]) sei nun über eine dritte durch fehlerhafte Ernährung verursachte, oft zum Tod führende Volkskrankheit berichtet, über den durch Jahrhunderte in manchen Regionen Europas Gesundheit und Leben gefährdenden Skorbut.

Mit der Frage, wie lang der Skorbut bereits bekannt ist, befasste sich besonders ausführlich der Medizinhistoriker August Hirsch (1817–1894) [3]. Er schrieb 1883 in seinem „Handbuch der historisch-geographischen Pathologie“: ob „Scorbut im Alterthume und im Mittelalter vorgekommen, bez. den Aerzten jener Zeit bekannt geworden ist, lässt sich aus den auf uns gekommenen Schriften jener Zeit nicht beurteilen“. Das Interesse der Ärzte der Neuzeit an dieser Krankheit wurde erst durch die Berichte über lang dauernde Entdeckungsfahrten zu See von Europa in andere Erdteile geweckt. Wer kennt heute noch den Skorbut außerhalb von Seefahrererzählungen? Wer erinnert sich, in historischen Romanen von vielen Belagerern und Belagerten gelesen zu haben, die plötzlich erkrankten und starben ohne einen Schwertstreich? Es war der auf beiden Seiten auftretende Scharbock, wie damals der Skorbut hieß. Und wer weiß heute, dass er noch im 19. Jahrhundert in manchen Gegenden eine gefürchtete Volkskrankheit war?

Noch bis ins 19. Jahrhundert wurde vielfach und unterschiedlich diskutiert, ob die von Ärzten des Mittelalters und der Neuzeit unter dem Begriff „Ignis sacer“, „Brunst“ [*Feuersbrunst*], „Prant“ oder „Brand“ geschilderten Krankheitsbilder nicht nur Ergotismus-, sondern auch Skorbutfälle waren. Für einige Medizinhistoriker war dies wegen mancher gleichartiger Symptome vorstellbar [3–7]. [Genaueres über Ignis sacer bei Flamm; [2]].

Conrad Heinrich Fuchs (1803–1855) bearbeitete „Das heilige Feuer des Mittelalters“ [5] insbesondere hinsichtlich dieser Differenzierung von Ergotismus und Skorbut. Der „Ignis sacer“ des 11. und 12. Jahrhunderts war nach seiner Überzeugung der Ergotismus. Aber der im 15. bis 17. Jahrhundert in „oft sehr mörderischen Epidemien auftretende“ Skorbut hatte z. T. ähnliche Symptome, nämlich heftige Schmerzen in den Extremitäten, insbesondere in den unteren, Ecchymosen und Ödeme an den Unterschenkeln. Die „bösartigen, häufig gangränescirenden Geschwüre entstehen aus diesen Flecken, und greifen selbst die Knochen an; die Sehnen der befallenen Glieder contrahiren sich, die Beweglichkeit erlischt, ohne dass die Schmerzen nachliesen, und in manchen Fällen sind die Unterschenkel abgemagert und so hart wie Holz“. „Allein nicht minder gross als die Analogien zwischen Scorbut und Ignis sacer sind die Differenzen zwischen beiden Krankheiten“. Die häufigsten Symptome des Heiligen Feuers [*Ergotismus*] sind „das Absterben und Abfallen [*!!!*] der Extremitäten vom Rumpfe“, während die besonderen Symptome des Skorbuts der Gingiva-Befall mit Ausfallen der Zähne und stinkendem Atem sowie große Müdigkeit und Kraftlosigkeit sind. „Und obwohl der Verlauf des Ignis sacer nicht akut war, scheint er sich doch nicht wie bei Scorbut Monate und Jahre lang hingezogen zu haben“. Auch die Orte der Verbreitung beider Krankheiten seien verschieden. „Der Scorbut findet sich vorzüglich auf und an der See, in niedrig gelegenen sumpfigen Gegenden, am liebsten in dem Bereiche miasmatischer Effluvien [*krankmachender Ausdünstungen*]; – die Küstenstriche von Holland und England, das russische Littoral der Ostsee und Dalmatien, waren daher der Schauplatz seiner größten Epidemien, und er ist endemisch an den Niederungen der Donau. – Das Heilige Feuer [*Ergotismus*] hingegen zeigte keine Vorliebe für solche Länder“; diese Krankheit ist „am häufigsten und mit den grössten Epidemieen erschienen“ in „Ostflandern, Lothringen, der gebirgigen Dauphiné und dem grössten Theil von Aquitanien, Isle de France und Leon“ und diese Regionen „sind weder Küstenstriche, noch Sumpfländer“.

Im 19. Jahrhundert wurde es also ziemlich sicher, dass der Skorbut und der Ergotismus zwei verschiedene Krankheiten sein müssen.

Die ersten verlässlichen Angaben der Symptome, die man für Skorbut halten kann, findet man nicht erst in Berichten von Seefahrern, sondern in der Schilderung des 6. Kreuzzugs (1248–1254) unter dem französischen König Ludwig IX., der selbst an Skorbut erkrankt war. Die Kreuzfahrer, welche die Belagerung von Damiette im Nildelta (1248) überlebt hatten, klagten über Verfall der Gingiva und Verlust der Zähne, wiederholte Schmerzen der Füße und Beine mit schrecklich schwärzlichen Flecken auf den Unterschenkeln. Im Laufe der Zeit wurden die Schmerzen heftiger und dauerten bis zum Frühjahr, bis die Wärme sie verschwinden ließ [8].

Die wahrscheinlich erste Mitteilung über „See-Skorbut“ bringt Johann Reinhold Forster in seiner „Geschichte der Entdeckungen und Schiffahrten im Norden“ [9] mit dem Bericht des Venezianischen Kaufmannes Pietro Quirini über dessen längere Irrfahrt in der Nordsee im Jahre 1431. Dieser berichtet: „Die Leute, welche bey der grossen Noth des Schiffes, alles für verlohren gehalten, und den schönen Malvasier Wein, den sie geladen, unmäßig getrunken hatten, die fielen, bey der zunehmenden Noth und überhandnehmendem Scharbocke, schnell und plötzlich todt dahin, dahingegen die mäßigen länger aushielten, und mit dem Leben davon kamen“.

Die europäischen Seefahrer ab dem 15. Jahrhundert, welche die küstennahen Gewässer mit ihrer steten Erreichbarkeit frischer Nahrungsmittel verlassen und auf lang dauernden Seereisen die anderen Kontinente erreicht hatten, mussten möglichst lange haltbare Lebensmittel mitführen. Das waren eingelegte Fische, Salzfleisch und Zwieback. Bei vielen Seeleuten entwickelte sich im Laufe der langen Fahrten eine tödliche Krankheit, die vielfach Skorbut in allen sprachlichen Formen dieses Namens genannt wurde. Meistens hatte die Erkrankung 12 bis 16 Wochen nach Beginn von langen Reisen begonnen. Als häufigste Symptome wurden Hinfälligkeit, Geschwülste der Schenkel mit Schwärze derselben, Krümmung und Unbeweglichkeit in den Gelenken, purpurfarbene Flecken und Blutungen über den ganzen Körper, faulendes Zahnfleisch mit Verlust der Zähne und aasartigem Geruch aus dem Mund angegeben. In den Logbüchern der Segler war die größte Sterblichkeit in den Monaten Jänner, Feber und März verzeichnet.

Das Ausmaß von Skorbut-Erkrankungen, die Besatzungen befallen konnten, zeigt der Bericht von Vasco da Gama (1460?–1524), der im Juli 1497 in Lissabon zur Entdeckung des Seeweges nach Indien aufgebrochen war. Auf dieser zweijährigen Fahrt hat er von den 160 Mann seiner beiden Schiffsbesatzungen etwa hundert verloren [8]. In diese Zeit fallen auch weitere größere Entdeckungsreisen, bei denen man auf den Skorbut als eine besondere Krankheit aufmerksam wurde.

In späteren Jahren häuften sich die Berichte über Skorbut bei Walfängern, die längere Zeit im Eis eingeschlossen waren oder durch widrige Winde zurückgehalten worden sind [3].

Im 18. Jahrhundert wurde das medizinische Interesse am Skorbut besonders durch das in drei Auflagen erschienene, viel beachtete und oft zitierte Buch „Treatise on the Scurvy in three parts“ (1753, 1757, 1772) des englischen See- und Hafenarztes James Lind (1716–1794) erweckt. Er betonte die Bedeutung der Krankheit durch die Feststellung, es gebe nur „wenige chronische Erkrankungen, die so schmerzhaft und mit solch einer Vielzahl von alarmierenden Symptomen behaftet sind, bei denen der Übergang vom Leben zum Tod oder von Krankheit zur Gesundheit so unerwartet und plötzlich eintritt; [*und*] die Entfernung der Ursache oft und beinahe unmittelbar eine Wirkung auf die Krankheit erzeugt“ [10].

## Der „Scharbock“ in der frühen Literatur

Die Bezeichnungen der Krankheit findet man erstmals im Jahre 1520 als „Scorbuck“ in der das normale Volksleben schildernden „Saxonia“ [11] von Albert Krantz (1450?–1517) und auch 1534 im „Botanologicon“ [12] von Euricius Cordus (1486–1535). Die Seefahrer der nördlichen Länder (Skandinavien, Niederlande, Norddeutschland) hatten in ihren Sprachen die Bezeichnungen für die Erkrankung gefunden. Zugrunde liegen die Begriffe für Bruch/Riss/Schrunde (*skjør (dän.), scheur (niederl.), scor, schor, schur und schar (dtsch.)*) oder aus dem Slavischen Scorb für Krankheit. Diesen Silben ist die Bezeichnung für ein betroffenes Organ angefügt: Mund: Scheurbek (ndl.), Schormundt, Scherer-Mund, Mund-Feule und Scharbok (dtsch.); Bauch: Sc(h)orbuc (dtsch.), Scheurbuik (ndl.); Knochen: Sc(h)orbein; Haut: Scurvy (engl., schuppig), Blauschuyt (ndl., blaue Flecken auf der Haut) [8, 10, 13–16]. Nach einer anderen Ableitung stammt der Begriff vom altnordischen skyrbjúgr [skyr = Sauermilch, Topfen, bjúgr = weiche Hautveränderung, also: Krankheit während der Zeit des Essens der Vorräte; [17]]. Noch im 19. Jahrhundert wurden in der deutschen Literatur die Bezeichnungen Skorbut und Scharbock ungefähr gleich oft gebraucht.

Als erste ärztliche Abhandlung der Neuzeit gilt die offenbar 1541 nur als Brief geschriebene „De Scorbuto, vel Scorbutica passione Epitome“ [18–20] des sehr geschätzten, aus niederländischer Familie stammenden Kölner Arztes Johann Bachoven von Echt (1515–1576), der nur als Johann Echt oder Ioannes Echt(h)ius in der Literatur aufscheint. Echt beschrieb die Krankheitserscheinungen des Skorbuts richtig und deren Unterschied zu denjenigen bei anderen Krankheitszuständen. Die Ursache des Skorbuts könnte eine Beeinträchtigung des Bluts sein, allerdings ohne Beteiligung der Eingeweide, insbesondere der Milz, der ja seit dem Altertum eine wichtige Rolle für Gesundheit und Krankheit zugeschrieben wurde. Außerdem sei der Skorbut auch infektiös.

Das literarische Problem war, dass Echts Epitome [*Zusammenschau, Übersicht*] offenbar nicht selbstständig publiziert worden ist. Um dies aufzuklären, haben die drei belgischen Ärzte Maurits Biesbrouck, Theodoor Goddeeris und Omer Steeno [19] den an verschiedenen Orten noch vorhandenen Briefverkehr bekannter niederrheinisch-niederländischer Ärzte aus Echts Umkreis sorgfältig studiert und verglichen. Im Jahr 2018 konnten sie berichten, dass die Epitome von Ioannis Echtius tatsächlich ab 1541 in Briefen im Kollegenkreis mitgeteilt, jedoch nie selbstständig publiziert worden ist. In Druck ist der Brief erst später viermal mit anderen Skorbut-Drucken gemeinsam der Öffentlichkeit bekannt gemacht worden.

Die von den drei Autoren reproduzierte Zeichnung des angeblichen Brustbildes von Echt ist insofern nur ein Symbol als in dem Werk von Pantaleon (1522–1595) [20], dem es entstammt, für die Angehörigen verschiedener Berufe des 16. Jahrhunderts sich wiederholende charakteristische Darstellungen gewählt worden sind. Es wurde das vermeintliche Bild Echts also auch für andere Ärzte verwendet.

Den ersten Abdruck der Epitome findet man in der 1. Ausgabe von „De magnis Hippocratis lienibus, Pliniiqve stomacace, ac sceletyrbe, seu vulgo dicto Scorbuto, Libellus“ von Balduinus Ronsseus (Boudewijn Ronsse, 1525–1597) [21]. Diese Ausgabe von 1564 enthielt nur die eigene Publikation Ronsses und die fälschlicherweise mit Ioannis Wierus als Autor angegebene Epitome Echts. Die 2. Ausgabe von 1585 [22] war umfänglicher. Nach Ronsses Text folgten die Epitome, nun richtigerweise unter Echts Namen, und die Texte von Ioannes Wierus (Johann Weyer, 1515–1588) und Ioannes Langius (1485-1565, Abb. 1).

**Abb. 1:**
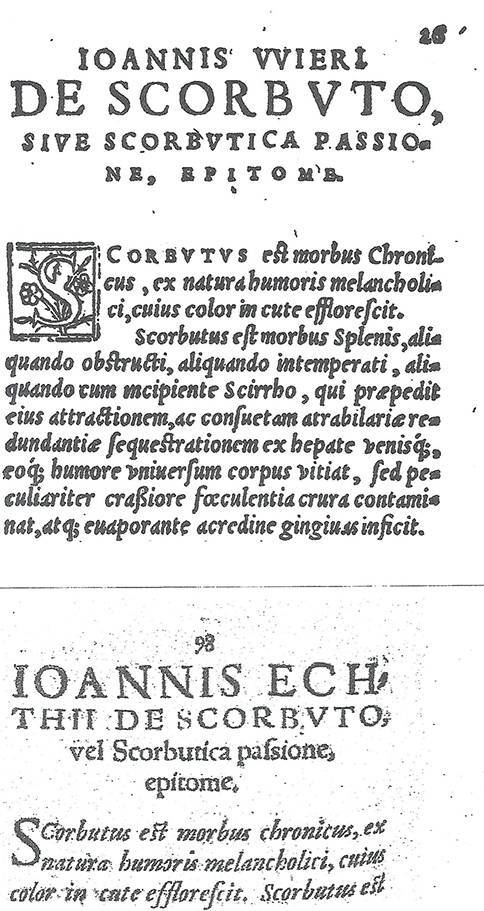
De Scorbvto, sive Scorbvtica Passione, Epitome. Als dessen Autor nennt Balduinus Ronsseus zuerst Ioannes Wierus und danach Ioannes Echthius in der 1. bzw. 2. Auflage von „Magnis Hippocratis Lienenibus … Libellus“ bzw. “Magnis ... Commentariolus” [21, 22]. Freundlicherweise zur Verfügung gestellt von der Österreichischen Nationalbibliothek [∗69.M.256(_3_)] bez. der Deutschen Forschungsgemeinschaft – Digitalisierung von Drucken des 16. Jahrhunderts

**Abb. 2:**
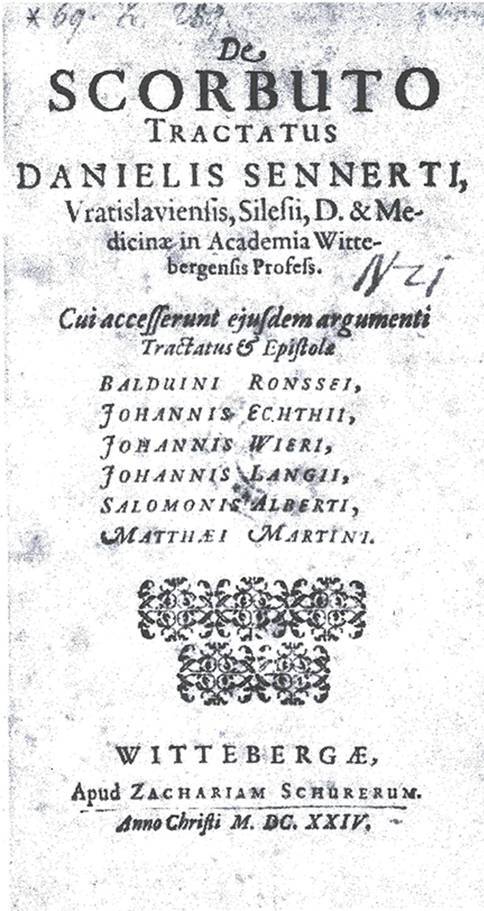
De Scorbuto Tractatus Danielis Sennerti [23]. Freundlicherweise zur Verfügung gestellt von der Österreichischen Nationalbibliothek [∗69.K.289]

Die nächsten zwei Publikationen von Echts Epitome enthalten die beiden Ausgaben von „De Scorbuto Tractatus“ [23, 24] des in Breslau geborenen und an der Universität zu Wittenberg lehrenden Daniel Sennert (1572–1637). Er fügte seinem eigenen Text einige weitere in seiner Zeit beachtete Bearbeitungen des Skorbuts an, nämlich jene von Balduinus Ronsseus, Johannes Echthius, Johannes Wierus [15], Johannes Langius [25], Salomon Albertus (1540–1600) [26], Ernestus Hettenbach (eine Dissertation unter Albertus) [27] und Matthæus Martinus (1587–1616) ([28]; Abb. 2).

**Abb. 3:**
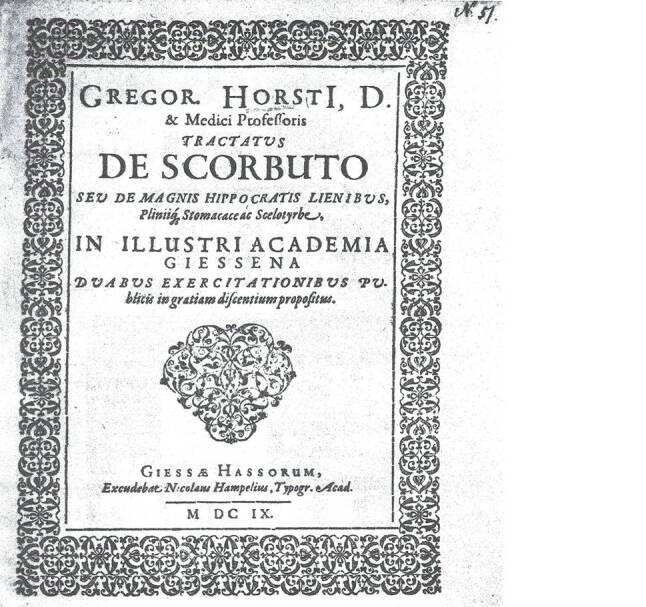
Gregor. Horsti Tractatus de Scorbuto. [29]. Freundlicherweise zur Verfügung gestellt vom Münchener DigitalisierungsZentrum

Um die Wende des 16. zum 17. Jahrhundert beschäftigte sich der in seiner Zeit sehr geschätzte, seine Tätigkeitsorte sehr oft wechselnde Gregor Horst (1578–1636) mit zahlreichen Krankheiten. Bezüglich des Skorbuts veröffentlichte er in den Jahren 1609 und 1615 den „Tractatus de Scorbuto“ [29] bzw. „Das Bůchlein von dem Schorbock/Gemeynem Vatterlandt zum besten Teutsch beschrieben“ ([30]; Abb. 3). Er verband mit reichlichen Zitaten antiker und auch zeitgenössischer Ärzte insbesondere die Erörterung der nach damaliger Meinung überragenden Bedeutung der Milz für Gesundheit und Krankheit, also auch für den Schorbock. Er gibt auch viele Rezepte für alle möglichen Erscheinungen der Krankheit.

Eine in ihrer Zeit sehr geschätzte Veröffentlichung war der im Jahre 1647 erschienene „Bericht und Vnterricht von der Kranckheit des Schmertz-machenden Scharbocks – Woher derselbe entstehet, komme und wie solche Kranckheit zu curiren“ [31] von Johann Drawitz (1604–1653). Dieser hatte seinen Bericht aufgrund von Mitteilungen über den 1486 zum ersten Mal in Sachsen grassierenden Scharbock verfasst. Dieses Werk war in seiner Zeit so wichtig, dass der Dekan der Leipziger Universität, Johann Michael, nach des Autors Tod auf Ansuchen des Verlegers sich hat „bewegen lassen/solches zu revidiren/und dem Nechsten zur Ersprießung [*im Jahre 1658*] in Druck wieder zu befördern“.

Unterdessen war der Skorbut bereits von akademischem Interesse geworden und als Thema von medizinischen Dissertationen an verschiedenen Universitäten. Der berühmte Jenaer Anatom und Schüler Sennerts, Werner Rolfinck (Guernerus Rolfincius, 1599–1673), erfasste im Jahre 1655 20 medizinische Dissertationen seiner Universität in seiner in Jena erschienenen „Epitome methodi cognoscendi & curandi particulares corporis affectus, secundum ordinem Abubetri Rhazæ ad Regem Mansorem libro nono, Hippocratis, Paracelsis & Harveanis principiis illustratæ et recognitæ, Philiatrorum in gratiam adornata & exscripta“ [16]. Enthalten ist darunter das Buch von Wilhelm Heinrich Schwartz „De Dolore Lienis, affectu hypochondriaco et scorbuto“ [32], in dem der Autor im Kapitel Skorbut bereits im ersten Satz auf die seit dem Altertum noch immer geglaubte ätiologische Bedeutung der Milz hinweist.

Unter dem Vorsitz Rolfincks sind bis 1669 zumindest vier weitere Dissertationen über Skorbut erschienen: 1640 von Hieronymus Bierling (?–1649) [33], 1648 von Laurentius Blumentrost (1619–1705) [34], 1668 von Johannes Loëlius (1641–1700) [35] und 1669 von Gottfried Schulz (1643–1698) [36].

## Die Erscheinungen des Skorbuts

Ursprünglich wurde die Krankheit ganz allgemein beschrieben. Sie beginne uncharakteristisch mit Mattigkeit und Müdigkeit, Druck auf der Brust mit Atembeschwerden, geschwollenem Gesicht, unerträglichen Kopfschmerzen, der Harn sei bisweilen „dick vnd weißlicht“. Wenn die Krankheit zunahm, würden diese Symptome heftiger und es käme zu Blutungen des Zahnfleisches und dessen Anschwellung und „Erfaulung“; die Folgen wären Zahnschmerzen, vermehrtes Ausspucken und stinkender Atem, bald Ausfallen der Zähne. Auch Nasenbluten könnte dazukommen. Schmerzen der Hüften und Beine erschwerten das Gehen bis zur Bewegungshemmung. Wegen der auch auftretenden Krämpfe und Lähmung von Beinen und Händen sprachen manche Ärzte von der Kriebelkrankheit^1^. Die an den Schienbeinen und oft auch an anderen Stellen entstehenden flohstichartigen Effloreszenzen könnten sich später zu größeren bläulichen bis schwarzen Flecken ausdehnen, aus denen sich bisweilen weiche Geschwülste und auch böse, kaum abheilende Geschwüre entwickeln. Zu diesen Erscheinungen kämen Durchfall oder Verstopfung, Fieber, Ohnmacht. Man sagte, der Skorbut wisse sich so zu verstellen, dass man auch an eine andere Krankheit oder an Zauberei denken könnte.

Ab dem 18. Jahrhundert untersuchte man den Verlauf des Skorbuts genauer.

Eine systematische Beschreibung des Skorbuts aus dem Wiener Bereich stammt von Gerhard van Swieten (1700–1772), dem Protomedicus^2^ von Maria Theresia. Er gliederte in seinen „Erläuterungen der Boerhaavischen Lehrsäze von Erkenntniß und Heilung der Krankheiten“ [37] die Entwicklung der Krankheit in vier Stadien.

Der Skorbut beginnt mit ungewöhnlicher Müdigkeit des ganzen Körpers, mit Trägheit und dem Verlangen zum Sitzen und Liegen wegen Schwierigkeiten beim Gehen und Stiegen steigen verbunden mit Muskelschmerzen.

Beim 2. Grad des Skorbuts wird das Atmen schwer und beschleunigt mit Atemnot bei kleinen Bewegungen. Die Beine schwellen zeitweise an und werden wegen ihrer Schwere unbeweglich, sie zeigen rote, braune, gelbe und bläuliche Flecken. Das Gesicht ist blass-bräunlich. Das geschwollene, heiße und schmerzende Zahnfleisch blutet bei geringem Drücken, es ist zurückgezogen von den Zähnen, die wackeln. Aus dem Mund strömt ein Gestank. „Verschiedene abwechselnde Schmerzen an allen äusserlichen und innerlichen Theilen des Cörpers, welche wundersame Schmerzen des Unterleibes, der Rippen, des Magens, der kleinen und grossen Gedärme, der Nieren, der Gallenblase, der Leber, der Milz u.s.w. hervorbringen, und verschiedenes, aber geringes Bluten.“

Beim 3. Grad des Skorbuts ist das Zahnfleisch brandig entzündet, es blutet und stinkt wie Aas. „Die wackelnden Zähne sind gelb bis schwarz verfärbt und haben Defekte. Die Froschader [*Vena ranina: der in der Zungenmuskulatur verlaufende Teil der V. lingualis*] zeigt knotige Ringe. Es treten öfters tödliche Blutungen aus „Mund und Nase, Lungen, Magen, Leber, Milz, Gekrösedrüse, Gedärmen, der Mutter [*Uterus*] und den Nieren u.s.w.“ auf. Hartnäckige brandige „mit langwierigem Gestank herumziehende Geschwüre“ treten besonders an den Beinen auf. „Das aus den Blutadern gezogene Blut ist an rothen Theilen schwarz, geronnen, dicke und doch aufgelöst, an wässerigten Theilen salzig, scharf und auf der Oberfläche mit gelbem grünem Schleim bedeckt, heftigst fressende, stechende, bald vorbeygehende, bey Nacht zunehmende Schmerzen in allen Gliedern, Gelenken, Knochen und Eingeweiden; blaue Flecken.“

Van Swieten berichtet aus eigener Erfahrung, „daß bey dieser Krankheit die flüßigen sowohl als festen Theile des Leibes dergestalt ausarten, daß die flüßigen Dinge, aus den Gefäßen, in denen sie enthalten sind, aus der geringsten Ursache heraustretten; wenn sie von der noch ganzen Haut des Cörpers eingeschlossen erhalten werden, jene rothe, himmelblaue, braune und blaue Flecken u.s.w. verursachen; ja daß man sogar unter den Zwischensätzen des musculösen Fleisches dergleichen Austrettungen des Blutes wahrgenommen habe. In einem solchen Fall aber verhindert das ausgetrettene geronnene Blut den ferner weiten Ausbruch oder Ausfluß des Blutes. Wenn aber dieses in solchen Gefäßen geschiehet, welche gegen die innerliche oder äusserlichhe Oberfläche des Cörpers zu offen stehen, so entstehen daraus nicht selten die gefährlichsten und oft ganz wunderbaren Verblutungen.“

Die an den Schienbeinen und insbesondere an den Knöcheln auftretenden Geschwüre sind von brauner bis purpurroter bleicher Farbe mit zerfressenen Rändern, aus denen ein stinkender blutiger Eiter fließt.

Im 4. Stadium des Skorbuts entstehen wechselnde oder anhaltende Fieber, Wassersucht, Erbrechen, Durchfälle, mit dem Stuhlabgang von Blut, Auszehrung, Zittern, Krämpfe, Lähmung, schwarze Flecken mit Nekrosen und nach damaliger Meinung schnelle Ansteckungen anderer Menschen.

Den üblen Gestank aus dem Mund von an Skorbut Erkrankten beschrieb besonders drastisch der in den Türkenkriegen bewährte ungarische Militärarzt und Kaiserliche Protomedikus am Rhein, Johann Georg Heinrich Kramer (?–1742) in seiner „Medicina Castrensis“, nämlich dass er „lieber zehen, so an denen Franzosen [*Syphilis*] oder auch an der Dysenterie darnieder liegen, als 5 Scorbutische in einem Zimmer haben will“ [38].

Die jeweiligen Kenntnisse der Ärzte des 16. bis 19. Jahrhunderts findet man in guten zeitgenössischen Überblicken zum Beispiel von James Lind (1716–1794) [10], Ludovic Rouppe (1729–1780) [39], Armin Langheinrich (1816–?) [40], Rudolph Krebel (1802–1865) [8] und Jean Bernhard Bornträger (1851–1927) [41], aber auch in Berichten weiterer Autoren.

## Das pathologisch-anatomische Bild des Skorbuts

**Abb. 4:**
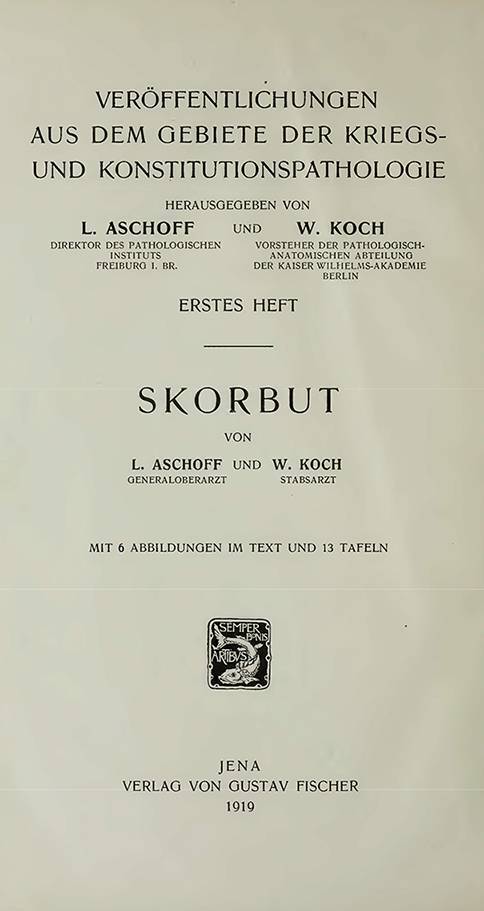
L. Aschoff und W. Koch: Skorbut. Gustav Fischer, Jena, 1919 [42]

Die pathologisch-anatomischen Befunde bei 23 Obduktionen von Soldaten des Winterfeldzuges in den Karpaten des Jahres 1917 beschrieben der Freiburger Pathologe Ludwig Aschoff (1866–1942) und sein ehemaliger Schüler Walter Koch (1880–1962) in ihrem umfänglichen Buch über „Skorbut“ ([42]; Abb. 4). Ergänzt werden hier deren Ergebnisse durch Angaben anderer Untersucher. Dazu gehören insbesondere auch die gemeinsamen Untersuchungen der japanischen Militärärzte Tatsujiro Sato (1868–1959) und K. Nambu an etwa 700 russischen Kriegsgefangenen nach Ende des japanisch-russischen Krieges im Jänner 1905 [43].

Äußerlich auffällig sind die vor allem an den Beinen, aber auch auf dem ganzen Körper auftretenden blutroten bis bräunlichen, in ihrer Größe variablen petechialen Hautblutungen. Daneben kommen auch subkutane fleckige und auch tiefer liegende größere Blutergüsse vor. Die Blutungen üben einen indurierenden Druck auf die umliegenden Gewebe aus. Haut und subkutanes Bindegewebe sind aber auch häufig ödematös, wie man auch Flüssigkeitsansammlungen in Herzbeutel, Bauch- und Brusthöhle findet.

**Abb. 5:**
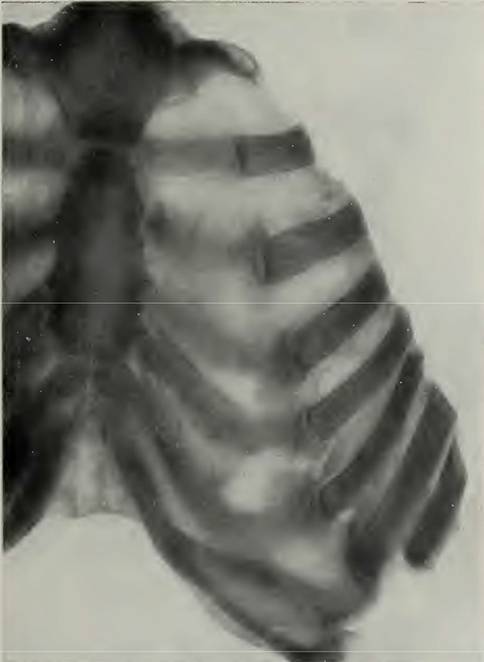
20-jähr. Soldat mit akutem schweren Skorbut. Völlige Fraktur der 3.–7. Rippe an der Knorpelknochengrenze mit Verschiebung der Rippenenden vor die Knorpelenden und gleichzeitiger Verschiebung gegen die Knochenknorpelachse. Aus Aschhoff und Koch, Abb. 37 [42]

Charakteristisch sind die Muskelblutungen vorwiegend der unteren Extremitäten. Sie infiltrieren Bindegewebe und Muskulatur, in der sie sich vorwiegend in den vorhandenen Gewebelücken ausbreiten. Die Blutungen sind sehr viel deutlicher in den oberflächlichen, den Sehnen und Aponeurosen der Muskelbäuche benachbarten Abschnitten als in deren Innerem. Blutungen unter dem Periost verursachen dessen Ablösung von den Knochen, vorwiegend an den Rippen und auch von der Vorderseite der Tibia. Die Blutungen im Knochenmark fehlen nie und sind bei schweren Fällen außerordentlich umfangreich. Sie kommen hauptsächlich an den Enden der Rippen im Bereich der Knochenknorpelgrenzen vor. Dort werden die inneren Knochenstrukturen zerstört. Es kommt zur Trennung von Rippen und Knorpeln. Das „Trümmerfeld“ der Rippe wird gegen den Knorpel hin durch Fibrin abgedeckt. Als Folge dieser Fraktur verdrehen sich Knorpel und Rippen unabhängig voneinander. Bei schweren Frakturen, bei denen auch die Corticalis der Rippen durch subperiostale Blutungen betroffen ist, kann das Rippenende bis auf die Hälfte der Knorpelbreite zusammengedrückt sein. Abb. 5 und 6.

**Abb. 6:**
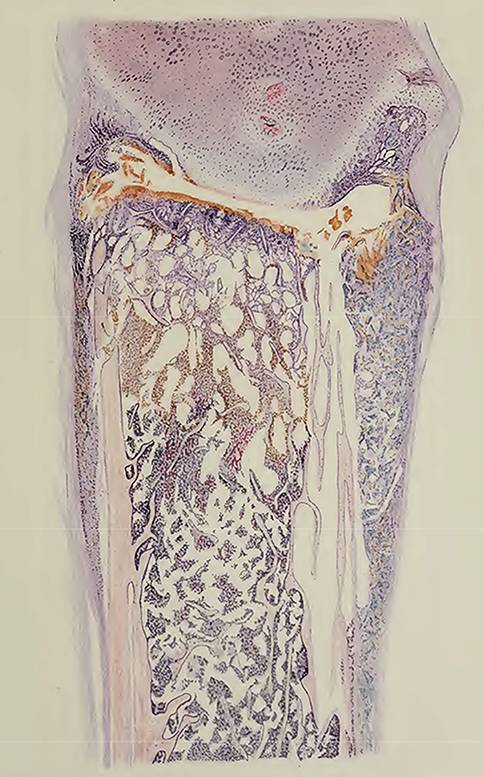
Derselbe Patient. Fraktur der 9. Rippe an der Knorpelknochengrenze mit Verschiebung des Knorpels nach außen. Ausgedehnte subperiostale Blutungen, besonders außen. Knochentrümmerfeld mit Fibrinbelag am Frakturspalt. Rarifikation der proximalen Knochenspangen. Aus Aschhoff und Koch, Abb. 24 [42]

Im Fett- und Bindegewebe um die großen Gefäße und Nerven treten ebenfalls diffuse Blutungen auf.

Die Zahnfleischveränderungen, die bei Skorbut-Beschreibungen meist besonders betont werden, sind zwar ziemlich häufige (~ 60 %), aber nicht immer auftretende Symptome des Skorbuts. Das dunkle bis schwarzrot gefärbte Zahnfleisch hebt sich von den Zahnhälsen ab, es blutet durch mechanische Einwirkung oft reichlich. Häufig ist es geschwürig zerfallen und mit einem schmutzigen, eitrigen Belag bedeckt. In den interdentalen Zwischenräumen bilden sich blutende Wucherungen. Diese zerfallen bald jauchig und stinkend, was wohl auf Bakterienbefall beruht. Dadurch werden die Zähne bis zum Rand der Alveolen entblößt und sind meist locker und wackelig. Der Gewebszerfall legt selbst die Kieferknochen auf weite Strecken frei. Die Schleimhautveränderungen können sich auch auf die Wangen und den harten Gaumen ausbreiten. Die peridentalen Gewebe sind empfindlich auf äußere Einwirkungen, wie z. B. das Kauen harter Nahrung oder bakterienbehaftete Schäden der Zähne. Dadurch entsteht der Zerfall des Zahnfleisches. Jene Bereiche des Kiefers von Erwachsenen (wie auch von Kindern vor dem Zahndurchbruch), die ohne Zähne sind, werden von den Veränderungen nicht betroffen [44].

Als ursächlich für die Lokalisation der Blutungen beim Skorbut werden mechanische Einflüsse angenommen. Für die Beine könnten Muskelbeanspruchungen beim langen Stehen oder durch Druck von Schuhwerk oder bestimmte berufliche Tätigkeiten infrage kommen, für die Rippen die Atembewegungen. Blutungen der Haut findet man dort, wo kräftige Muskulatur Bewegungen ausführt, an den Schenkeln, am Gesäß und an den Unterarmen, während Hände, Füße und Gesicht mehr frei bleiben. Am Herz fehlen trotz seiner ständigen Muskelbewegungen skorbutische Blutungen.

## Der Landskorbut

Ab der Mitte des 15. Jahrhunderts erschienen verschiedene lokale Nachrichten aus dem nördlichen Europa bis hinauf aus dem hohen Norden über „Landskorbut“. Aber noch über weitere Jahrhunderte galt Gerhard van Swietens Feststellung, dass es allen mit dem Scharbock befassten Ärzten sehr schwerfällt, diese Krankheit zu diagnostizieren und die zur Unterscheidung charakteristischen Zeichen anzugeben [37]. So sind also manche Berichte mit Vorsicht zu lesen.

Nach 1478 soll der Skorbut begonnen haben sich epidemisch auszubreiten. So werden die in einer Chronik aus Meißen für das Jahr 1486 geschilderten „Entzündungen der fleischigen Teile“, die bei Fehlen rascher Hilfe in Brand übergegangen sind, von manchen Autoren als Skorbut aufgefasst. Ab Mitte des 16. Jahrhunderts findet man dann Angaben über den Skorbut in der chronischen Form besonders im Norden [8, 45]. So berichtete 1655 etwa der Erzbischof von Upsala, Olaus Magnus (1490–1568), in seiner geschichtlichen Abhandlung über die Völker im Norden, dass besonders die Dänen und Niederländer und überhaupt jene, die in der Nähe von Küsten leben, vom Skorbut, vulgo Schoerbuck, betroffen seien [46].

In derselben Zeit wurde über das epidemische Auftreten der Krankheit auch in den Bevölkerungen der Niederlande und Norddeutschlands, des Baltikums, Frankreichs, Lothringens und Genfs, auch von Böhmen, Mähren, Schlesien und Schwaben berichtet. Besonders in den Niederlanden wurde das lokale Auftreten des Skorbuts betont, der in „niedrigen, sumpfigen Provinzen“ häufig vorkam, während er in deren benachbarten Gegenden ziemlich unbekannt war [47].

Nach der Häufigkeit der Veröffentlichungen scheint der Skorbut Im 19. Jahrhundert in Europa endemisch verbreitet gewesen zu sein. Er trat in den tiefer liegenden Küstenregionen und sumpfigen Landstrichen auf. Die Berichte betreffen Grönland, Großbritannien, Norwegen, Schweden, Dänemark, Spanien, Nordfrankreich, Holland, Friesland, Niedersachsen, die Gebiete entlang der Donau, Russland, die Küsten Italiens, des Schwarzen und des Kaspischen Meeres. Zusammenfassend also waren es die Gegenden, die, wie man sagte, „feucht“ waren. Für die Jahre 1556 bis 1877 hat der Medizinhistoriker August Hirsch 120 Skorbut-Epidemien des europäischen Festlandes und weitere 23 bei in Übersee eingesetzten Truppen europäischer Staaten zusammengestellt. Aber auch aus Asien, Afrika und Amerika gibt es Berichte über „Landskorbut“. In gemäßigten und in kalten Zonen trat der Skorbut meistens im Winter und Frühling auf [3].

In den unterschiedlichen Gebieten des großen Habsburgerreichs, das von den Gipfeln der Alpen über die Karpaten bis zu den Bergen Transsilvaniens mit den umschlossenen Ebenen des Marchfelds und des Alfölds [*Ungarische Tiefebene*] reichte, bewohnt von seinen zahlreichen, zum Teil noch mit alten Vorstellungen behafteten Völkern, sind lokale Krankheitsausbrüche lange Zeit unerkannt geblieben. Dazu hat nicht zuletzt auch die mancherorts mangelhafte medizinische Versorgung, insbesondere außerhalb der Städte beigetragen.

Ein Beispiel für die Aufdeckung und die Umstände einer Skorbut-Epidemie in einer Bevölkerung stammt vom Protomedicus von Ungarn, Franz von Schraud (1761–1806), in seinem Bericht über die Epidemie des Jahres 1803 in drei Bezirken des Temescher Banats [48].

Er berichtet von einem Offizier, der zu Ende Hornung [*Feber*] 1803 seine Truppe von der Reichsgrenze nach Temesvar [*Temeschburg, Timişoara*] zu überstellen hatte. Beim geplanten Anmarsch zu einem Ort für die Einschaltung eines Rasttages erfuhr er, dass dort täglich 7 bis 8 Leichen begraben werden, weswegen er einen Umweg nahm. In Temesvar war des Offiziers Bericht für das Generalat [*Militärbehörde*] und die Gespanschaft [*königlich-ungarische Komitats-Behörde*] die erste Anzeige der „bereits seit längerer Zeit dagewesenen und weit umher verbreiteten Krankheit“. Beide Institutionen „machten ungesäumt alle Anstalten, um Gewisheit über das Daseyn und verlässlichen Bericht über die Natur des entdeckten Uebels zu erhalten“. Es begaben sich der Arzt der Gespanschaft, der Regimentsarzt und die Wundärzte von Gespanschaft und von einigen königlichen Kammergütern an Ort und Stelle.

In den gemeinsamen Untersuchungen erkannten sie „an den gesamten Kranken den Scharbock, welcher sich allenthalben durch blasses, aufgedunsenes Gesicht; verdorbenes, lockeres, leicht blutendes Zahnfleisch; Gestank, und Blutung aus dem Munde; wankende, oder ausgefallene Zähne; beschwerliches Kauen; durch grosse, zu Tag, wie Nacht, gleich starke Schmerzen in den oberen und unteren Gliedmassen, in der Schultergegend, und Halsmuskeln; durch eigene Niedergeschlagenheit, das Gefühl der Ermüdung und Hinfälligkeit; durch blaue Flecke von verschiedener Grösse und Gestalt; durch verhärtete Geschwülste in beyden Extremitäten; durch, oberflächliche Geschwüre unverkennbar darstelle und auszeichne, wozu bey manchen sich Fieberbewegungen gesellten, welche nach keinem bestimmten Typus gerichtet waren.“

In der Folge fanden die genannten Ärzte und Wundärzte auch in den anderen besuchten Orten solche Kranke und bald kamen die entsprechenden Anzeigen von mehreren Gemeinden, „welche nun der öffentlichen Aufmerksamkeit entsprechen zu müssen glaubten“. Bei der genauen Durchsicht der örtlichen Totenlisten „fand man meistens in den Monathen Jenner und Hornung eine mehr als gewöhnliche Sterblichkeit“.

Man hatte auch bemerkt, dass nirgends Angehörige der deutschen Sprachgruppe betroffen waren, auch dort nicht, „wo Deutsche gemeinschaftlich, und in was immer für welchem Verhältnisse vermengt mit Walachen^3^ wohnten“. Nur unter letzteren waren Skorbutkranke zu finden, aber auch das nicht in allen walachischen Ortschaften. Am meisten betroffen waren Orte nahe bei beiden Ufern des Flusses Temesch, nahe an Reisfeldern oder Sümpfen. Männer erkrankten weniger häufig als Frauen und Jugendliche beider Geschlechter. Die Erkrankung wurde weder während der Schwangerschaft noch durch die Muttermilch auf die Kinder übertragen.

„Indem die Gesundheits-Beamten von der Aufzeichnung der Kranken zum Heilgeschäfte derselben, zur Wahl der gehörigen Arzneymittel, zur Bestimmung des nöthigen Heilverfahrens übergingen: trafen sie auf Schwierigkeiten mancher Art, mit welchen der praktische Arzt in Städten bey der Behandlung einzelner Kranken viel seltener zu kämpfen hat, deren einige ihm [*sogar*] ganz unbekannt sind“. „Zwar sind die Heilmethoden gegen den Scharbock im Allgemeinen so richtig im voraus bestimmt“, doch zur Anpassung an die einzelnen Verhältnisse der Kranken hatte der Gespanschafts-Arzt noch zu wenig Angaben, um den untergeordneten Wundärzten Weisungen erteilen zu können. „Der vorhergehende Gesundheits-Stand des Volkes“ im Banat [*in dem die betroffenen Gespanschaften lagen*] war zu wenig bekannt „und konnte nur einigermassen aus dem Gange der Krankheiten“ in den betroffenen Orten „errathen werden“. „Der Gang des Scharbocks selbst war zu wenig beobachtet“, um dessen Richtungen „in den Tod oder [*zur*] Besserung zu kennen“ und somit individuelle Vorschriften für das „Benehmen der Wundärzte bey einzelnen Kranken“ festsetzen zu können.

„In noch grössere Verlegenheit wurde der Arzt versetzt“, der hier und „bey dem Mangel aller hierauf sich beziehenden Polizey-Vorschriften“ selbst handeln musste. Und dies wurde „durch die Rohheit des Volkes“ ganz besonders erschwert, das durch Unwissenheit, Eigensinn und Widerspenstigkeit die nötigen Maßnahmen nicht annahm. Aus „blinder Anhänglichkeit und Vertrauen an widersinnige Gebräuche“ sah die Bevölkerung monatelang unbekümmert und sorglos dem Elend und dem Absterben seiner Hausgenossen zu. An den Grabstätten wurde sie dann „durch die Tollheit geschüttelt“, die „Ursache des grossen Uebels in Vampyren zu finden“. Die Menschen fielen „rasend über längst verscharrte Leichen her“ und „misshandelten die Todten, um desto beruhigter für die Lebenden nichts zu thun, desto beherzter und zuversichtlicher jeder dahin gehörigen Aufforderung zu widerstehen“. Der Vampirismus war im Volk allgegenwärtig.

Aus diesen Gründen war es angezeigt, für das Heilverfahren sehr wenige und nur einfache Mittel einzusetzen. Fast allgemein wurden dazu mit Kren [*Meerrettich*] versetztes Bier und auch Senf in Zwetschken [*Pflaumen*]-Brand verabreicht. Gegen die Leiden des Mundes diente Spülen mit Salbei-Aufgüssen mit Alaun, Salzsäure, Honig und auch Myrrhentinktur; zum Waschen der starren Extremitäten wurden warmer Essig oder Kampfergeist verwendet.

Für mehrere benachbarte Orte wurde jeweils ein Wundarzt bestellt, der diese möglichst oft besuchen musste. Dessen Aufgabe war, das bisherige Auftreten der Erkrankungen und den Verlauf der einzelnen bis dahin ungefähr 4000 Erkrankungen in den betroffenen drei Bezirken sowie die Wirksamkeit der Behandlungen zu erforschen. Dazu halfen mehrere in jedem Ort bestimmte Geschworene, die bei ihren täglichen Hausuntersuchungen neue Krankheitsfälle und die Zustände der bereits Erkrankten festzustellen hatten. Zur Verteilung der im Gemeindehaus gelagerten Arzneien wurde ein eigener Mann beordert und für deren Verteilung wie auch für andere von den Wundärzten angeordnete Dienste wurden Männer aus der ärmeren Klasse bestimmt, die sog. Krankenwärter, Dorfdiener oder Kleinrichter. Der Gespanschafts-Arzt versammelte alle vierzehn Tage die Gesundheits-Beamten jedes Bezirks zur Berichterstattung.

In seinem Bericht kommt Franz von Schraud „näher zur Quelle der unter den Walachen beobachtete Krankheit“. Die Nahrung im Winter beruhte auf Mais in Form von Brei oder Brot, dem es „gewöhnlich an guter Zubereitung gebricht“. Dazu kamen frisches, auch eingesalzenes oder geräuchertes Schweinefleisch, luftgetrocknete Fische und als „einziges Zugemüse“ Sauerkraut. Im zu Ende gehenden Winter litten die walachischen Bauern durch Mangel an Nahrungsmitteln, insbesondere an pflanzlichen. Der Skorbut zeigte sich überall, wo Sauerkraut fehlte, nicht aber in Gegenden, wo dieses erzeugt wurde oder durch gedörrte Zwetschgen ersetzt werden konnte. Als weitere Ursachen zieht von Schraud die schlechten Verhältnisse vieler walachischer Häuser in Betracht. Die „Vorbereitungs-Ursache des Skorbuts“ wird „ungemein verschlimmert, oft tödlich“, durch das viele und strenge Fasten der walachischen Bevölkerung. An den jährlich 238 Fasttagen sind Fleischspeisen verboten, an vielen auch noch Eier, Milch, Schmalz und Butter. „Der in dieser Enthaltsamkeit selige Beruhigung findende Mann ist auf äusserst wenige, schwer verdauliche, unschmackhafte Nahrung beschränkt.“ Diesem Übel begegnete er mit einem Übermaß geistiger Getränke, was die Gesundheit weiter untergräbt.

Franz von Schraud hat aus seinen Beobachtungen und Erfahrungen bei dieser eigentlich erst durch einen Zufall entdeckten lokalen Skorbut-Epidemie den Vorschlag von „Vorschriften der medizinischen Polizey für nicht ansteckende Volkskrankheiten“ entwickelt und als Buch veröffentlicht [48].

Die schon von Olaus Magnus [46] 1555, wohl auf Grund der Berichte aus der Kreuzfahrerzeit geäußerte Feststellung, der Skorbut sei ein Morbus castrensis qui vexat obsessos et inclusos, also einer Lager-Krankheit, die Belagerer und Eingeschlossene verheert, bestätigte sich auch in späteren Zeiten für die österreichischen Truppen.

So berichtete Johann Georg Heinrich Kramer im Jahre 1720 in seinem „Consilium de Scorbuto inter militem Cæsareum“ der Wiener Medizinischen Fakultät seine Erfahrungen über Skorbut bei in Festungen belagerten k. k. Truppen. Er beobachtete bei der Besatzung in einem belagerten Ort, dass das in Schwaben rekrutierte Regiment im Gegensatz zu den anderen aus Böhmen stammenden Regimentern frei von Skorbut geblieben ist. Dies führte er auf die unterschiedlichen Diäten der Soldaten zurück. Seinen Bericht beendete er mit dem Wunsche, ein verlässliches Mittel kennen zu lernen, durch das der Skorbut verhütet oder geheilt werden kann [49].

In ihrer Antwort [50] bestätigt die Fakultät, dass nach sicheren Beobachtungen der Skorbut nur im Frühjahr beginnt, in Italien und im Banat aber nur die ankommenden kaiserlichen Truppen ergreift. In Deutschland bekämen einzelne Personen den Scharbock auch in gesunden Orten, wenn sie selten oder nie Kräuter und Obst genießen. Daher grassierte diese Krankheit auch in lange belagerten, sonst gesunden Städten. In den insgesamt 73 Punkte umfassenden Erörterungen behandelt die Fakultät ausführlich das Bild der Krankheit.

Johann Georg Heinrich Kramer betonte übrigens in seiner umfänglichen, „allen Feld-Medicis und Feldscherern teutscher Nation zum Besten“ gewidmeten Monografie „Medicina Castrensis“ [38] die Bedeutung der Ernährung. Es erkrankten nämlich „nur gemeine Soldaten und keine Officiers“ am Skorbut. Dies führte er auf deren bessere, auch Vegetabilien enthaltende Ernährung und gesündere Unterbringung zurück.

Der Begründer der k. k. Chirurgischen Militärakademie in Wien und erster Protochirurgus des gesamten k. k. Militärs, Johann Alexander von Brambilla (1728–1800), berichtete über den Ausbruch von Skorbut in der in Schlesien stehenden Armee [51]. Die Soldaten waren sehr schlecht untergebracht, sie lagen auf dürftigem Stroh oder direkt auf dem bloßen Boden und wegen der Teuerung aßen sie weniger Sauerkraut und tranken weniger Bier als bisher. Im Dezember 1761 trat Skorbut vermehrt auf und bis im folgenden März entstand „eine so gefährliche und tödtliche Seuche, die mehr Menschen [*ergriffen hatte*] als der Feind hätte aufreiben können“. In manchen Regimentern lagen zwei Drittel der Soldaten im Spital. Dagegen blieb die Armee in Sachsen, deren Quartiere und Nahrung besser waren, bis zur Vereinigung der beiden Armen verschont.

Als die Armeen Anfang April 1762 in das Lager einrückten, mussten die Unterwundärzte und Unteroffiziere „gute und essbare Kräuter“ wie Brennnesseln, Kresse, Zichorien, Borresch und Ampfer suchen; welche die Soldaten mit dem Fleisch kochen mussten. Für die Absonderung der Scharbockkranken wurde hundert Schritte hinter der Regiments-Ubikation eine Hütte errichtet, die zur Ermöglichung einer guten Belüftung an allen vier Seiten Fenster und Türen hatte. In dieser wurden die Kranken nach den damaligen Methoden behandelt.

In der vom Deutschen Bund (1815–1860) ab dem Jahre 1840 langsam ausgebauten Bundesfestung Rastatt lagen auch k. k. Truppen. Im Jahr 1852 betrug ihre Stärke 4300 Mann. An Skorbut erkrankten vom k. k. Linien-Infanterie-Regiment 602 Mann und von den anderen Truppenteilen acht Soldaten, insgesamt also 14 %. Von den Erkrankten starben 25 Mann. Unter den Soldaten der Badischen Strafkompanien und der armen Zivilbevölkerung in und um Rastatt kam der Skorbut nur sporadisch vor. Abgesehen von den zum Teil sehr ungünstigen Verhältnissen der Unterbringung der Soldaten hielt man auch die Alimentation als Teil der Ursache des Skorbuts. Da Gemüse und Hülsenfrüchte in dieser Gegend nicht in hinreichender Qualität vorhanden und außerdem sehr teuer waren, lebte die Mannschaft fast täglich von Suppe mit Rindfleisch und Knödeln. Dies betraf besonders die böhmischen Infanteristen, die, des Deutschen nicht mächtig, in der Freizeit die Unterkünfte kaum verließen, während die badischen Soldaten bei Verwandten und Freunden in der Stadt essen konnten und so von Skorbut frei blieben. Der damals behandelnde k. k. Regimentsarzt Eduard Opitz hält auf seine Erfahrungen zurückschauend diese Epidemie hinsichtlich der Menge der Erkrankten, der Mannigfaltigkeit der Erscheinungen und des Verlaufs für vergleichbar mit den zahlreichen von englischen und russischen Ärzten beschriebenen derartigen Ereignissen [52].

So wie in belagerten Städten kann es auch in anderen mehr oder weniger geschlossenen Personengruppen zum Auftreten von Skorbut kommen. Ein Beispiel dafür gibt der Bericht von 1859 aus dem Wiener Versorgungshaus am Alserbach [53]. Ab Feber oder März eines jeden Jahres zeigten sich bis Juli ansteigende Zahlen von Skorbut bei den Pfründnern. Erklärt wird dies damit, dass diese im Winter kaum lüften und „hindurch eine mit allerlei Effluvien geschwängerte Zimmerluft einatmen, die bei dem Zimmerwohnen von mehreren Menschen, wovon jeder an irgend einem Gebrechen leidet, mehr schädliche Potenzen enthalten dürfte als in solchen Localitäten, die mit gesunden Individuen angefüllt sind, wie z. B. in Casernen“. Als genau beschriebenes Beispiel dafür dient der Bericht des bekannten Militärsanitätshistorikers und späteren Generalarztes Salomon Kirchenberger (1848–1924) über die im April 1873 in der Prager Garnison ausgebrochene Skorbutepidemie [54].

Wirklich geschlossene Personengruppen lebten in den Straf- und „Correctionshäusern“, in denen noch im 19. Jahrhundert „selten ein ganzjähriger Zeitraum vorübergeht, ohne dass der Scorbut entweder selbstständig oder als Complication anderer Krankheiten zum Vorschein käme“ [55]. Das große Ausmaß, das die Erkrankung in manchen Jahren erreichen konnte, zeigt die von Johann Joseph Čejka (1812–1862) genau bearbeitete Epidemie des Jahres 1843 im Prager Provincialstrafhaus [56]. Von 777 Sträflingen waren 397 vom Skorbut ergriffen.

Der Grund hierfür könnte vermeintlich nur „in der Individualität der Sträflinge oder in der Natur der Strafe selbst liegen“. Schwerlich wird den Ärzten aus ökonomischen Gründen die für richtig gehaltene Behandlung möglich sein, „diese Krankheit erfordert nämlich, wenn sie epidemisch auftritt, keineswegs so wie der Seescorbut, frische Pflanzennahrung, sondern tägliche Fleischkost“. Aber andererseits zeigte die Erfahrung, dass auch die Verabfolgung von Milch „nicht nur als Prophylacticum, sondern auch als Heilmittel des Scorbuts die vorzüglichsten Dienste“ leistet. Als Prophylaxe wurde auch vorgeschlagen, die Gefängnisse „durch die Entlassung der minder gravirten Sträflinge und durch Entsendung der Gefangenen zur Feldarbeit“ „zu entvölkern“ [57].

## Der Morbus Möller-Barlow

In Bevölkerungen, in denen Skorbut häufig beobachtet wurde, war zu erwarten, dass auch Kinder von dieser Krankheit befallen werden. Es fehlten jedoch mit wenigen Ausnahmen durch lange Zeit spezielle Berichte darüber.

Kenneth John Carpenter (1923–2016) wies 1986 in seiner historischen Übersicht auf das häufige Auftreten von Skorbut in den Jahren 1590 bis 1640 in französischen karitativen Institutionen für vernachlässigte Kinder [58]. In England beschrieb im Jahre 1650 Francis Glisson (1598/99–1677) in seinem Buch „A Treatise of the Rickets being a Disease Common to Children“, dass mit Skorbut gemeinsam auch Rachitis auftritt und er dies als Folge mangelhafter Ernährung der Kinder betrachtet [59].

Am 14. Jänner 1778 berichtete der in Wien an Volkskrankheiten arbeitende Karl Ritter von Mertens (1737–1788) in einem Schreiben an die Royal Society of London (dort vorgetragen am 7. Mai d. J.) unter dem Namen Charles de Mertans über die nach seiner Berufung als Arzt nach Russland bereits in den Jahren 1767 bis 1772 gemachten Erfahrungen mit dem häufig vorkommenden Skorbut [60]. Insbesondere beschreibt er genau die Krankheit bei Kindern im Moskauer Waisenkrankenhaus. Viele wurden kachektisch und nahmen eine bleierne Farbe an, ihr Zahnfleisch schwoll zunehmend an und wurde livid, im Mund bildeten sich Pusteln, es wurden Gingiva und Mundschleimhaut gangränös und die Kieferknochen kariös, sodass die Knochen der Alveolen und die Zähne ausfielen und aus dem Mund stinkender Atem strömte; die Kinder konnten sich kaum bewegen, hatten aber kein Fieber und guten Appetit. Bei manchen waren die Beine geschwollen und bedeckt von skorbutischen Flecken und trockenen Krusten. Es wurden die Flektoren der Beine kürzer und steifer, sodass die Kinder eine liegende Position mit angezogenen Beinen (Frosch-Haltung) einnahmen. In zwei Fällen betraf dies auch die Arme. Die Zustände nahmen mehr oder weniger rasch zu und schließlich starben die Kinder. Wenn die Veränderungen nicht zu fortgeschritten waren, gab de Mertans pflanzliche Kost wie Sauerkraut, Karotten, Weiße Rüben, Zwiebeln, Sauerampfer. Als Getränke gab es Wasser oder saures leichtes Bier und Quas [*Getränk aus gegorenem Brot*]. Im Frühling wurden auch Molkegetränke mit antiskorbutischen Pflanzen wie Löffelkraut, Brunnenkresse oder Sauerampfer verabreicht. Zum Mundspülen dienten Wässer mit Heilkräutern wie Odermening, Gartenraute oder Salbei und auch Rindendekokte. Mit diesen Behandlungen konnte de Mertans gute Erfolge erzielen. Die Ursache der Erkrankung der Kinder, die er für Skorbut hielt, sah er in der Ernährung und in der zumindest zeitweise schlechten Unterbringung in feuchten Räumen.

Eine etwa gleiche Krankheitsbeschreibung wurde auch im österreichischen Kaiserreich festgestellt und von Johann Peter Frank (1745–1821), dem Begründer der Volksgesundheitslehre, in lateinischer Sprache im Discursus Academicus am 20. Mai 1788 an der Mailänder Akademie als „Rachitis acuta“ beschrieben und der „Rachitis adultorum“ gegenüberangestellt [61]. Frank gab an, dass diese Krankheit, soweit er weiß, bisher von niemandem beschrieben worden ist.

Entgegen der Publikation von de Mertans setzte sich Franks Bezeichnung „Rachitis acuta“ vorerst durch. Dabei ist Julius Otto Ludwig Möller (1819–1887) besonders zu nennen, da sein Name später in die Bezeichnung der Krankheit aufgenommen wurde [62]. Er publizierte in den Jahren 1859 und 1862 in Königsberg, Ostpreußen, die Symptome von fünf Kindern, die ihn an Rachitis denken ließen. Da jedoch im Gegensatz zum lang dauernden Verlauf der Rachitis sich die beschriebene Krankheit der Kinder sehr rasch entwickelte und zum Tode führte, verwendete Möller den Namen „Acute Rachitis“. Die durch die „Knochenblutungen entstehende Aehnlichkeit mit Scorbut“ hielt Möller „für eine rein äusserliche, symptomatische“.

In der englischen Literatur wurden die Untersuchungen von Walter Butler Cheadle (1836–1910) in seinem Londoner Kinderspital von 1878 oft zitiert [63]. Rachitis war dort die häufigste Krankheit, während Fälle von Skorbut bei Erwachsenen selten waren. Cheadle beschrieb drei Kinder im Alter von 16 und 36 Monaten, bei denen zum bekannten Bild der Rachitis noch die charakteristischen Symptome des Skorbuts dazukamen. Dafür hielt er das Fehlen von Kartoffeln und Milch in der sonst üblichen einseitigen, vorwiegend aus Mehlprodukten bestehenden Kost für ursächlich.

Cheadle wies auch noch auf einen anderen wesentlichen Aspekt hin. Ausgeprägter Skorbut bei Kindern in großen Städten war selten. Er meinte jedoch, dass die Fälle von ulzerierender Stomatitis, die man oft bei schlecht ernährten, vernachlässigten Kindern in der Stadt fand, eine Folge der „skorbutischen Konstitution“, also „imperfekt entwickelter Skorbut“ sein könnten.

Eingehender befasste sich 1884 Thomas Barlow (1845–1945) im Londoner Kinderkrankenhaus mit der „akuten Rachitis“ anhand von klinischen Befunden und Sektionsprotokollen [64]. Besonders charakteristisch waren subperiostale Blutungen in den Spalt zwischen den Epiphysen der langen Knochen und den Gelenksknorpeln, welch letztere sogar verschwunden sein konnten. Es gab auch Blutergüsse in den tiefen Muskeln. Die Rippenknochen waren von ihren Knorpeln getrennt. In den Knochen fanden sich charakteristische Veränderungen: Ihr Gewebe war aufgelöst, sodass nur eine äußere „Schale“ übergeblieben ist, was die Frakturen erklärte.

Diese Befunde bei dieser vorerst als „Osteopathia haemorrhagica infantum“ bezeichneten Krankheitsform unterschieden sich deutlich von jenen bei gewöhnlicher Rachitis, glichen aber genau jenen, die Lind für Skorbut beschrieben hatte. Die „neue“ Krankheit fand man auch bei Kindern, „denen jedes Zeichen von Rachitis fehlt und die unter günstigen Verhältnissen leben“. Selbst bei ernstesten Fällen von Rachitis gab es aber „kein Symptom dieser neuen Krankheit“. Es fehlten die subperiostalen Blutungen, die charakteristischen Veränderungen in der Mundhöhle und auch die Kachexie. Besserung und Heilung waren durch antiskorbutische Behandlung sowohl lokaler Art (Kompressen, Bäder, wenig Bewegungen) als auch durch Ernährung mit rohem Fleischpresssaft, frischer Milch, Orangensaft und rohem frischem Gemüse zu erreichen. Die „Akute Rachitis“ sei also eine Kombination von Skorbut und Rachitis, wobei „der Skorbut ein wesentliches und die Rachitis ein variables Element ist“.

Barlows Erkenntnis war also, dass die „neue Krankheit“ ein Skorbut ist, der sich durch die größere Häufigkeit des Knochenbefalls vom Skorbut der Erwachsenen unterscheidet. Man soll daher in Hinkunft von „kindlichem Skorbut“ und nicht von Akuter Rachitis sprechen.

Es wurde jedoch bereits in den 1890er-Jahren der Namen „Barlow’s Disease“ oder „Barlowsche Krankheit“ auch im deutschen Sprachraum weitgehend allgemein verwendet. Aber bald erinnerte man sich der Veröffentlichung von Möller und so schrieb 1896 Harald Hirschsprung (1830–1916) in Kopenhagen über „Die Möller’sche Krankheit (Synon.: Acute Rachitis. Scorbut bei Kindern. Barlow’sche Krankheit. Cheadle-Barlow’sche Krankheit etc.)“ [104]. Im Ersten Weltkrieg führte man, wohl aus patriotischen Gründen, den heute gebräuchlichen Namen „Möller-Barlow-Krankheit“ ein [58].

Aus vielen Berichten ist bekannt, dass der kindliche Skorbut nie bei brustgestillten Kindern zu beobachten war. In diesem Sinne steht auch die Tatsache, dass Skorbut in Russland trotz seiner weiten Verbreitung in der Bevölkerung kaum bei Kindern auftrat, da diese üblicherweise an der Brust gestillt wurden [65]. Genau das Gegenteil stellten viele Untersucher fest, die in wirtschaftlich besser gestellten Familien ohne Skorbut der Erwachsenen bei deren Kindern die Barlow’sche Krankheit feststellen mussten. Es war nämlich oftmals üblich, die Kinder nicht an der Brust zu stillen, sondern mit industriell gefertigten Lebensmitteln zu füttern. Kinder aus ärmeren Klassen, die an den gewöhnlichen Mahlzeiten der Erwachsenen teilgenommen hatten, blieben in der Regel frei von Skorbut.

Untersuchungen aus 1984 zeigten, dass die Konzentration des skorbutverhütenden Stoffes (Vitamin C) in der Muttermilch im Vergleich mit dessen Gehalt im mütterlichen Blutplasma bis zu achtmal höher sein kann [66].

Genaueres über die verfütterte Milch untersuchte 1902 Hugo Neumann (1858–1912) in Berlin in einer umfänglichen Studie [67]. Sie zeigte, dass die Krankheit bei allen Kindern aufgetreten ist, die mit sterilisierter oder doppelt pasteurisierter Milch gefüttert worden sind, nicht aber wenn im Haushalt gekochte Milch verwendet worden ist. Nach vielen physikalischen und chemischen Versuchen mit Milch kam er zur Meinung, dass der durch Erhitzung wahrscheinlich gebildete toxische Stoff durch Reaktionen des Milcheiweißes entstanden ist. Die damalige Häufigkeit der Krankheit in Berlin sei also auf die Erhitzung der Milch im Soxhlet-Apparat zurückzuführen.^4^ Er betonte die Wichtigkeit, die Ernährung auf rohe oder vorsichtig pasteurisierte Milch umzustellen. Alfred Fabian Hess (1875–1933) fand, dass das Alter der Milch wichtiger zu sein scheint als die Art der Erhitzung, dass sogar rohe Milch durch Altern die antiskorbutische Wirkung verliert [68].

Die Diskussion, ob die Akute Rachitis oder Möller-Barlow’sche Krankheit wirklich ein typischer Skorbut ist, beschäftigte weiterhin viele Untersucher. So bearbeitete 1912 Karl Hart (1876–1922) in Berlin diese Frage in Versuchen mit Rhesusaffen [69]. In seiner Publikation „Über die experimentelle Erzeugung der Möller-Barlowschen Krankheit und ihre endgültige Identifizierung mit dem klassischen Skorbut“ schrieb er: „Es ist mir zum ersten Male geglückt, nachzuweisen, daß beim Skorbut der Erwachsenen und [*bei der*] Möller-Barlow-Krankheit die gleichen Knochenveränderungen auftreten und damit den letzten endgültigen Beweis für die Identität beider Affektionen zu erbringen. An Stelle des nichtssagenden Verlegenheitsausdruckes ‚M.-B. K.‘ sollte man nur von ‚kindlichem Skorbut‘ sprechen“, eine Meinung, die im Jahr davor auch auf der 73. Versammlung Deutscher Naturforscher und Aerzte in Hamburg [70] geäußert worden ist. Harts „gewonnener Gesamteindruck“, dass für die Entstehung des kindlichen Skorbuts eine individuelle Disposition eine sehr bedeutende Rolle spiele, wurde offenbar durch seine Affenversuche bestätigt. Denn bei einseitiger Verfütterung von im Handel erhältlicher kondensierter Milch^5^ an zehn „noch nicht ausgewachsene“ Rhesusaffen erkrankten im Beobachtungsjahr einige Tiere schon bald schwer, andere erst später, unter dem Bild, das sich „vollkommen mit der menschlichen M.-B. K. deckt“. Die Ursache sah Hart nicht in der Milch an sich, sondern in der einseitigen Ernährung.

Mit der Frage der Milchaufbereitung befasste sich etwa zur selben Zeit auch Theodor Frølich (1870–1947) in Oslo [71, 72]. Meerschweinchen entwickelten bei Fütterung von Hafer mit gekochter oder noch stärker erhitzter Milch stets Skorbut, war jedoch die verfütterte Milch roh oder nur bis 70^o^C erwärmt worden, so blieb der Skorbut aus. Frølich schloss aus diesem Verhalten auf einen thermolabilen Inhaltsstoff der Milch, der Skorbut verhütet.

## Ursachen des Skorbuts

Die vielen Meinungen über die Entstehung des Skorbuts und die mehr oder weniger zufälligen Erfahrungen mit seiner Heilung und Vermeidung änderten sich im Laufe der Jahrhunderte. So können sie in ihrer Entwicklung nur mehr oder weniger gemeinsam besprochen werden.

Das primäre Interesse am Skorbut ging von der Seefahrt aus. In Berichten der frühen Seefahrer liest man auch vom Erfolg des Verzehrs von grünen Pflanzen und deren Wurzeln, Knollen, Zwiebeln und von rohen oder gekochten Kartoffeln. In nördlichen Gebieten, wo solche pflanzliche Nahrung fehlte, fand man im Genuss von Koniferennadel-Abkochungen und von rohem Fleisch und rohen Organen von Meeressäugetieren eine wirksame Hilfe gegen Skorbut.

Neben diesen praktischen Erfahrungen gab es theoretische Grundlagen für die Erklärungen der Ursachen des Skorbuts. Es war lange Zeit der noch aus dem Altertum stammende Glaube an die „Sex res naturales“ [*Res: Sache, aber auch Umstand, Ereignis*]. Das waren Luft, Ernährung, Verdauung, körperliche Betätigung, Schlaf und frohes Gemüt. Nach dieser Lehre sollten zur Verhütung des Skorbuts dazu neigende Menschen „ein diæt, die dem Schorbock zuwider ist“ einhalten. Dabei bedeutet „Diät“ in diesem Gebrauch die zur Wahrung der Gesundheit notwendigen Vorkehrungen gegen alle den Leib und das Leben bedrohenden exogenen und endogenen Gefahren.

Dementsprechend diskutierte Gregor Horst (1578–1636) in seinem „Büchlein von dem Schorbock“ anfangs des 17. Jahrhunderts unter Zitierung antiker Autoren die nach seiner Meinung notwendigen Maßnahmen zu Verhütung und Heilung des Skorbuts, nämlich „eine warme/truckene/vnd dem wesen nach reine Lufft“, gute Nahrung, mäßige Bewegung, „Reinigung der vberflůssigen sachen“ zur Verhinderung der „Samlung bōser feuchtigkeiten“, kein unmäßiger Schlaf, keine langwierige Traurigkeit und keine melancholischen Gedanken [29, 30].

Wichtig erschien den Ärzten dieser Zeit, die Verhinderung der Entstehung schädlicher „Feuchtigkeiten“ im Körper und deren Bekämpfung zu beachten. Es sollte also der Medicus zur „Curatio Scorbuti“ nach vorhergehender milder Purgation den Patienten zur Ader lassen, damit aus dem Magen und den Eingeweiden die Feuchtigkeiten von den Adern an sich gezogen werden. Dadurch bestehe die Hoffnung, dass sich „die Miltz und beyliegende Glieder“ selbst leichter ihrer „vnreinigkeit“ einiger Maßen entledigen könnten als viele böse Feuchtigkeiten nur durch den Stuhl abgehen zu lassen. Für die Purgation wurden Rezepte für etliche „Tråncke“ und Pulver angegeben. Zur Entfernung der Feuchtigkeit müssen auch Harn- und Schweißproduktion gefördert werden. Sie verlässt aber den Körper insbesondere durch die „außdempffung der důnste“ durch die „Schweyßlōcher“ der Haut. Auch Bäder befördern dies.

Wenn die Krankheit trotz Behandlung weit fortgeschritten ist, müsse man insbesondere auf „Mundt, Schenckel und beyne“ achten. Gegen die Mundfäule wirken zusammenziehende Mundwässer mit Lorbeerblättern, Eichenlaub, Schlehdornrinde und anderen pflanzlichen Produkten, aber auch mit Korallen, Alaun [*Kaliumdoppelsulfat*] und Vitriol [*wasserlösliche Schwermetallsulfate, bes. von Cu, Fe, Zn*]. Bei Beteiligung von Hüften und Schenkeln muss man vor allem für Kontrakturen und Lähmungen wie auch für Flecken, Geschwülste und Geschwüre besondere Arzneien einsetzen. Hüften, Schenkel und Beine badet man außerdem in Kräuterwässern. Geschwüre behandelt man mit Arzneien, die reinigen und zusammenziehen.

Die „Sex res naturales“ finden wir, wenn auch nicht dezidiert erwähnt, als Grundlage der weit verbreiteten Suche nach den Ursachen des Skorbuts der Seeleute auf langen Fahrten. Dafür machte man das Leben an Bord verantwortlich mit seinen Unterkünften in feuchten, schlecht gelüfteten Räumen, mit überanstrengender Arbeit, psychischen Belastungen durch das gedrängte Leben auf engem Raum mit wenigen Ablenkungen in der Freizeit und fehlender Körperreinigung. Die Ernährung, die üblicherweise aus gesalzenem Fleisch und Fisch, Zwieback, Brot, Hafermehl, getrockneten Erbsen, Käse und Butter („Diaeta grossa et corrupta“ [47]) bestand, spielte nach mancher Meinung eine untergeordnete Rolle. Selbst gänzlicher Mangel an Gemüse und Früchten in der Kost soll nicht zwangsläufig Skorbut verursacht haben. Nach der Meinung mancher Ärzte löste der Mangel an Gemüse und Früchten nur dann Skorbut aus, wenn prädisponierende Umstände wie eine kalte, nasse Atmosphäre herrschten, welche die Poren des Körpers blockierten. Trotzdem war die allgemein verbreitete Meinung, dass Skorbut vermieden und geheilt werden könnte, wenn man nicht nur grüne Pflanzen in die Nahrung aufnimmt, sondern auch Früchte, insbesondere Zitrusfrüchte [73].

Für den Skorbut auf dem flachen Land gilt als wesentliche Ursache neben dem Leben in Behausungen mit schlechter Luft auch das Essen von roher, grober und unverdaulicher Nahrung und das Trinken ungeeigneter Getränke. Im Einzelnen werden angeführt: kleiehaltiges, geräuchertes, schimmeliges Brod; Rind‑, Bock- und Schaffleisch, wenn sie alt, trocken und mager sind; auch geräucherte Fische; Federvieh aus feuchten und sumpfigen Orte; ferner Sauerkraut, Bohnen und Erbsen aus feuchten Orten und alter Käse. Auch „dicker grober“ wie auch wässriger unreiner Wein mit viel Weinstein sowie mit wenig Hopfen gebrautes Bier wurden genannt. Vom Wasser hieß es, je schwerer, gröber und unreiner es ist, desto mehr verursacht es den Scharbock, gleichgültig ob man es trinkt oder zum Bierbrauen verwendet. Durch Essen und Trinken, aber auch durch andere Sachen kann die übermäßige Feuchtigkeit vermehrt werden.

Der Wunsch nach Vermeidung von Nahrungsbestandteilen aus feuchter Umgebung zeigt wieder die Furcht vor zu viel Feuchtigkeit. Diese erschien berechtigt, fand man doch den Skorbut gehäuft, auch oft epidemieartig, in Orten am Meer oder in sumpfigen Gegenden. Dies galt auch für oft überschwemmte oder von der Tide betroffene Orte. Daher war in Ländern wie Holland, Friesland und Dänemark die Krankheit sehr verbreitet. Als Beispiele diente das besonders starke Auftreten von Skorbut, nachdem die holländischen Küstenregionen im Jahr 1569 von großen Überschwemmungen und deren Folgen (Nahrungsmangel, zerstörte Behausungen) heimgesucht worden waren. Auch die Hungersnot des Jahres 1573 in Holland verursachte durch die mangelhafte Ernährung Skorbut und entvölkerte ganze Dörfer.

Unmäßigen Schlaf aber auch vieles Wachsein würden die Krankheit heftiger machen, insbesondere wenn ein trauriges Gemüt dazukommt. Dadurch entstünde dann eine Sammlung großer „melancholischer Feuchtigkeiten“.

Zur Entstehung des Skorbuts trüge auch das Fehlen der gewöhnlichen Reinigungen bei, insbesondere bei Ansammlung von „unreinem Blut“ im Körper, wie wenn bei alten Männern „die gewõhnliche Gůlden ader^6^ verstopfft“ oder bei Frauen „jhre Monatzeit verstopfft“ ist.

Von manchen Ärzten wurde auch die Frage diskutiert, ob der Skorbut vielleicht auch angeboren sein kann. Man meinte, die „Schorbutische schwachheyt der Miltz wirdt von den Eltern den Kindern mitgetheylet/sonderlich durch den Samen/welcher rohe vnnd verderbet ist“. Es gelte also, „daß ein Vatter mit dem Schorbock behafftet/den Kindern solche Kranckheyt anerbt“. Franz von Schraud verfügte jedoch über Berichte, dass bei skorbutkranken Schwangeren kein Abortus beobachtet wurde, sondern gesunde Kinder geboren wurden. Es wurden auch von solchen Müttern gesäugte Kinder nicht von der Krankheit befallen [48]. Um etwa 1800 verstummten derartige Fragestellungen.

Immer wieder wurde auch behauptet, dass der Skorbut eine „anklebende“ [*ansteckende*] Seuche sei, denn es wären Leute, die lang und viel mit solchen Patienten beschäftigt waren, wenn auch oft erst nach langer Zeit, „beflecket worden“. Auch die Beobachtungen der Häufigkeit der Krankheit in Verbindung mit der Gepflogenheit des Küssens bei der Begrüßung und des gemeinsamen Trinkens aus einem Gefäß, insbesondere in den Niederlanden und in den Hansestädten, schien ein Hinweis auf die Infektiosität des Skorbuts zu sein. Die Frage blieb noch bis ins 19. Jahrhundert nicht sicher geklärt.

## Die Plantae antiscorbuticae

In manchen Bevölkerungen des Europäischen Festlandes war bekannt, dass einige wild wachsende Pflanzen gegen Skorbut besonders gute Dienste leisten. Diese und in der Folge etliche weitere Pflanzen wurden zur Therapie und Prophylaxe verwendet.

**Abb. 7:**
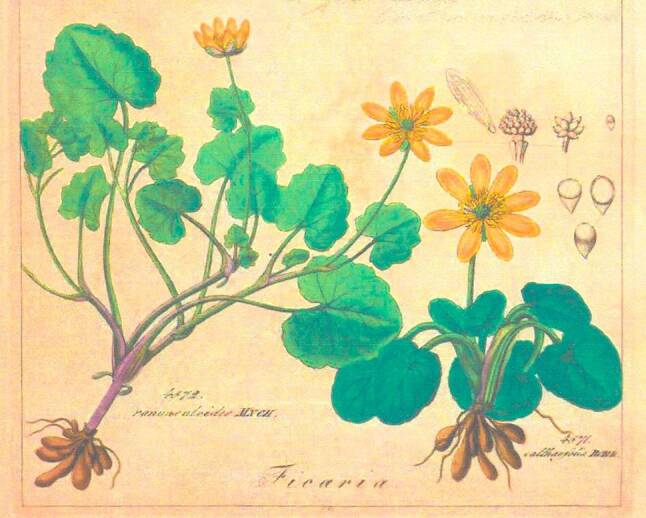
Scharbockskraut (Ficaria verna = Ranunculus ficaria). Aus Reichenbach, Vol. 3: Kap. Ranunculaceae Tafel I [79]. Freundlicherweise zur Verfügung gestellt von der Österreichischen Nationalbibliothek [http://data.onb.ac.at/rep/10A630EC]

Von den „Plantae antiscorbuticae“ [74] wurden vorzugsweise die nach heutiger Terminologie als „Scharbockskraut“ (Ficaria verna = Ranunculus ficaria), „Großes Schöllkraut“ (Chelidonium majus) und „Löffelkraut“ (Cochlearia officinalis) bezeichneten Pflanzen verwendet (Abb. 7, 8 und 9).

**Abb. 8:**
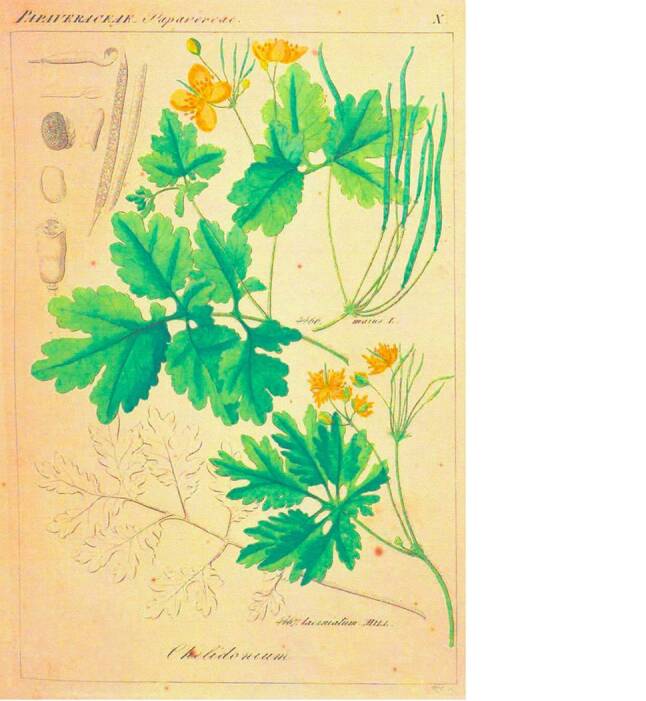
Großes Schöllkraut (Chalidonium majus). Aus Reichenbach, Vol. 3: Kap. Papaveraceae Tafel X [79]

**Abb. 9:**
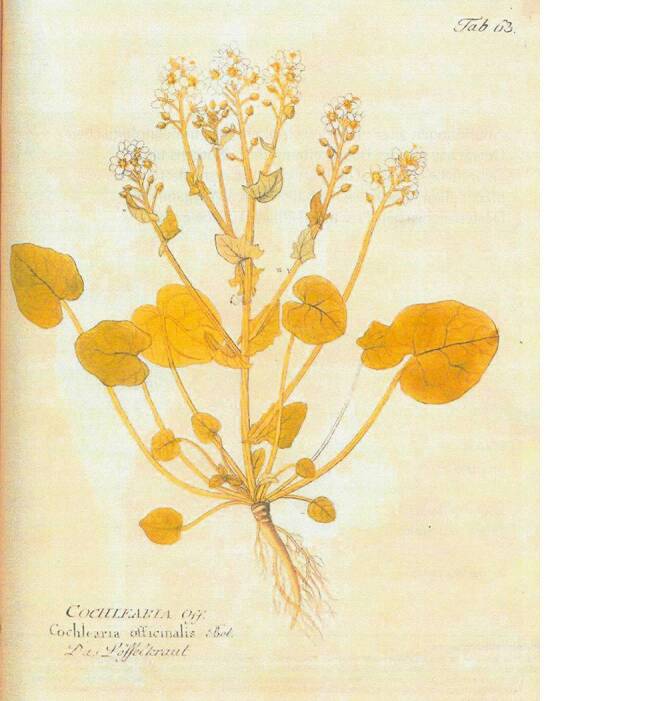
Löffelkraut (Cochlearia officinalis). Aus Vietz: 1. Theil, Tafel 63 [76]. Freundlicherweise zur Verfügung gestellt von der Österreichischen Nationalbibliothek [http://data.onb.ac.at/rep/10501865]

Erstere, die den sprechenden Namen Scharbockskraut tragende Blütenpflanze eignete sich besonders zur Verhütung und Bekämpfung des Skorbuts, da sie zeitig im Frühjahr als eine der ersten Pflanzen aus der Erde sprießt, sich jedoch nach der Blüte bald zurückzieht. Wichtig war die Kenntnis, dass die Blätter ab der Blüte giftig sind.

In der frühen medizinischen Literatur werden die vielen gebräuchlichen Namen zum Teil verwirrenderweise auch wechselseitig verwendet. Euricius Cordus (1486–1535) bezeichnete 1534 in seinem Botanologicon [12] das „Große Schöllkraut“ als „meykraut“ [*Mai-Kraut*] und auch „feickvuartzen kraut“ [*Feigwarzen-Kraut*] und berichtete, dass es die Sachsen dagegen „schorbocks kraut“ nannten.

Fünf Jahre später verwendet Ioannes Agricola (1496–1570) in seinen „Medicinae Herbariae Libri duo“ [75] für das Chelidonium ebenfalls die Namen „Große Schöllkraut“ und „Mai-Kraut“, aber für die Ficaria die Bezeichnungen „pfaffen hödlin“ [*Pfaffen-Hoden*] und „feigwurtz“.

Weitere Namen für das „Scharbockskraut“ (Ficaria) verwendete 1818 der erste Professor für Staatsarzneikunde der Universität Wien, Ferdinand Bernhart Vietz (1772–1815), in seinem zehnbändigen Werk „Abbildungen aller medizinisch-ökonomisch-technischen Gewächse mit der Beschreibung ihres Nutzens und Gebrauches“ [76] nämlich „Feigwarzen-Ranunkel“ [*Ranunculus* *=* *Hahnenfuß*], „Scharbocksranunkel“ und sogar „Kleines Schöllkraut“ [*wohl weil das „Scharbockskraut“ viel niedriger wächst als das „Große Schöllkraut“*].

Bei der Vielfalt dieser und noch weiterer verwendeter antiskorbutischer Pflanzen und deren vielen Synonymen fällt das 1674 erschienene Buch „Cochlearia curiosa, cum figuris et indice locupletissimo“ [77] des Jenaer Professors Valentin Andreas Moellenbrock (1623–1675) auf. Er konzentriert sich auf das Löffelkraut mit dessen vermeintlichem Wirkungsmechanismus und brachte viele Rezepte für die äußerliche und innere Anwendung. Die Wichtigkeit dieser Publikation beweist die bereits nach zwei Jahren erschienene englische Bearbeitung durch Thomas Sherley (1638–1678) mit einer Vermehrung der Rezepte [78].

Moellenbrock war wohl aus einer alchemistischen Vorstellung davon ausgegangen, dass in skorbutischen Säften des Kranken, aber auch in geräuchertem und getrocknetem Fleisch ein starkes und kompaktes „Salz“ vorhanden sei. Dieses sei beim Skorbut meist sauer und verursachte deshalb die unerträglichen Schmerzen. Wenn dieses Salz „volatil“ [*flüchtig*] gemacht wird, sei es abgeschwächt und könne durch die feinen Poren der Haut ausgestoßen werden. Durch das volatile Salz des Löffelkrauts würde es entgiftet und zerstört. Die hauptsächliche „Antiskorbutkraft“ dieser Pflanze sei nach der „Meinung beinahe aller [*damals*] modernen Ärzte“ aber „verborgen und mysteriös und unbekannt“.

Da das Salz des Löffelkrauts volatil sei, kann nur wenig gewonnen werden. Es genügte aber, dass sein „Spirit“ [*Geist*] verwendet werde, in dem das Salz versteckt enthalten sei. Dieses volatile Salz gewönne man durch Auspressen der kurz in Wasser gekochten dicken, saftigen Blätter des Löffelkrauts. Noch besser sei es, die frisch gesammelten Blätter auszupressen und den gewonnenen Saft einzudampfen, bis er dicklich wird und sich das volatile Salz absetzt. Betont wird, dass Löffelkrautblätter nur stets frisch gesammelt zu verwenden seien, weil anderenfalls das volatile Salz bei der Zubereitung bereits verschwunden sei.

Einen guten Überblick über die heimischen in der Medizin verwendeten und anderen Blütenpflanzen bietet das mehrbändige Werk „Icones Florae Germanicae et Helveticae“ von Ludwig Reichenbach [79].

## Therapie und Prophylaxe des Skorbuts

Ab etwa dem 15. Jahrhundert findet man Angaben über Behandlung und Vorbeugung des See- und Landskorbuts. Einen schönen Überblick über die Therapie von 88 Patienten in der Zeit um 1680 bringt Maximillian Mayer 2012 in seiner Würzburger Dissertation [80] mit den aus dem Lateinischen transkribierten Aufzeichnungen des Ulmer Stadtarztes Johannes Frank (1649–1725).

Im militärmedizinischen Bereich berichtete 1735 Johann Georg Heinrich Kramer (1684–1744) in seiner bereits oben erwähnten „Medicina Castrensis“ [38] unter anderem über „das einzige Præservativ vom Scorbut zur See“, nämlich „daß alle Schiffe eine quantité Sauerkraut mitführen und ihren Botsknechten wöchentlich ein paarmahlen austheilen lassen. Item daß die mit Scorbutischen behafften Schiffe nach Portugall eilen und mit dem Safft dasiger Pomeranzen ihre Kranken curiren“. Auf die gute prophylaktische Wirkung des zusätzlichen Einsatzes von Sauerkraut in der Verköstigung seiner Besatzung vertraute auch der englische Weltumsegler James Cook (1728–1779) auf seiner zweiten Seereise 1772–1775. Bei seiner Rückkehr konnte er berichten, durch diese Kost keinen seiner 112 Seeleute an Skorbut verloren zu haben.

Den Versuch einer experimentellen Untersuchung der Ernährung von Seeleuten unternahm James Lind im Jahr 1747 als Arzt an Bord von HMS „Salisbury“ [10]. Er teilte 12 skorbutkranke Seeleute in sechs Zweiergruppen, denen er zur Kost verschiedene Heilmittel der damaligen Zeit verabreichte, nämlich Cider [*Most*], Vitriol-Elixir [*Vitriol-Tinktur*], Essig, Seewasser, Knoblauch-Electuarium [*musartige Zubereitung*] oder Orangen und Zitronen. Nach zwei Wochen erwies sich das zuletzt genannte Paar als geheilt. Die anderen Paare waren unverändert oder nur insignifikant gebessert.

James Lind empfahl danach 1753 in seinem Buch „Treatise on the Scurvy in three parts“, neben Citrus-Säften zusätzlich auch Essig zu verabreichen. Den seefahrenden Ärzten riet er, an Bord auf Textilien als Unterlage Senfkraut und Kresse anzubauen und davon den Seeleuten tägliche Rationen zu geben.

Aus seiner Erfahrung wusste Lind, dass ein an Bord fortschreitender Skorbut bei Seeleuten, die für einige Tage an Land gebracht worden sind, stark und plötzlich gut beeinflusst wurde. Nach seiner Meinung schienen die Freude, nach langer Reise an Land zu sein, die Aussicht auf rasche Erlösung vom Elend an Bord, die Veränderung von Luft und Wetter und sogar die Wärme eines angenehmen trockenen Bettes die Wirkung anderer therapeutischer Anwendungen auf die Krankheit stark und überraschend zu übertreffen.

Lind hatte aber auch an Land in seinem Spital beobachtet, dass bei manchen mit der üblichen Kost an Bord gesund gebliebenen Seeleuten sich erst während ihres Aufenthalts im Hafen trotz Konsums von reifem Obst und grünem Gemüse erste Symptome von Skorbut einstellten.

Die Meinungen über die Wirkung von Obst und grünem Gemüse waren noch immer nicht einheitlich. So zögerte die englische Admiralität nach Linds 1753 veröffentlichen Ergebnissen noch vierzig Jahre, bis sie 1795 Zitronensaft an die Seeleute ausgab, wodurch der Skorbut sofort ausblieb [73]. Später wurde auf den Kauffahrtei- und Kriegsschiffen vieler Staaten eingeführt, dass zwei oder drei Wochen nach Beginn der Seereise an jeden Mann täglich etwa 20 g Zitronensaft in Wasser oder Rum abgegeben werden [81].

Kurz nach Linds Buch gab Maria Theresias Protomedicus Gerhard van Swieten in Wien seine „Erläuterungen der Boerhaavischen Lehrsäze von Erkenntniß und Heilung der Krankheiten“ heraus, in denen er die Therapie des Skorbuts ausführlich behandelte [37]. Man glaubte also, dass die Körpersäfte mit dem Fortschreiten des Skorbuts immer „schärfer“ würden. Bei der Auswahl der „antiscorbutischen Arzeneyen“ habe man deswegen die verschiedenen Stufen der Krankheit zu beachten, aber auch die verschiedenen Temperamente der Patienten. Es ist dabei auch „der Mangel solcher Nahrungsmittel, welche aus dem Pflanzenreiche genommen werden, unter die Ursachen des Scharbocks“ zu rechnen. In der Materia Medica nennt van Swieten als „besonders wider den Scharbock dienende Mittel“ Brühflüssigkeiten vom Sauerampfer, von den ersten Sprösslingen der Großen Klette, vom Roten Kohl, vom Körbelkraut [*Kerbel, Anthriscus cerefolium*], von Cichorien [*Wegwarte & Endivie*] oder [*Brenn*]Nesseln sowie die Säfte von Orangen und Zitronen.

Beim 2. Grad des Skorbuts empfiehlt er zusätzlich den „Gebrauch schärferer Scharbockspflanzen, in Form eines ausgepreßten Saftes, [*eines*] Eingemachtens, Geistes [*alkoholischer Extrakt*], flüchtigen weinigten [*Wein-*]Salzes, oder Arzeneybiers; desgleichen äusserliche Bäder und Fußbäder von Scharbockskräutern; warmes trockenes Reiben mit besonders würkenden flüßigen Mitteln; oft wird eine Aderläße nützlich seyn, damit der scharfe Theil der Säfte weggenommen, das Auffressen in denen zu sehr ausgedehnten Gefäßen verringert, das Zuruckziehen zuwege gebracht, und denen anzuwendenden Arzeneyen der Weg gebahnet werde“. Die in der Materia Medica als schärfer und sehr scharf angegebenen Pflanzen sind: „der indianische Kreß [*wahrscheinlich die Virginische Kresse, das Lepididium virginicum*], der Knoblauch, die Zehrwurz [*Gefleckter Aronstab, Arum maculatum*], Pfefferkraut [*Senfkresse, Satureia hortensis*], Mauerpfeffer [*Sedum acre*]“.

Wenn bei fortschreitender Krankheit „Zeichen einer größeren Schärfe und meistentheils auch der anfangenden Fäulniß zum Vorschein“ gekommen sind, werden die Fieber öfters hitzig und „die Gefäße von der Schärfe der Säfte zerfressen“, wodurch sich „Verblutungen“ einstellen. In der Folge können „die Säfte dergestalt aufgelöst“ sein, „daß sie nicht mehr in ihren Gefäßen können zurückgehalten werden“. Da „pflegen kluge Aerzte eine andere Art antiscorbutischer Mittel zu verordnen; solche nämlich, welche die festen Theile befestigen und stärken, und die allzudünnen Säfte wieder verbessern. Dergleichen Mittel sind Mengelwurz [*Arisaema triphyllum, ein Aronstabgewächs*], Engelsüß [*Polypodium vulgare, ein Tüpfelfarn*], Kappern und Tamariskenholzrinde, Sauerampfer“ und dergleichen aus der Materia Medica. Wenn die Symptome zweifelhaft sind oder eine Fäulnis noch nicht vorhanden, aber zu befürchten ist, werden „sogenannte kalte antiscorbutische Mittel“ gebraucht, wie Löffelkraut, Brunnenkresse, Sauerampfer, Zitronen und Orangen. Die Übel des Mundes werden lokal mit hitzedämpfenden Mitteln behandelt, wie Theriakgeist^7^, Löffelkrautgeist oder mit Campher versetzter Brandwein.

Wenn man beim 3. Grad der Krankheit in „unvorsichtiger Weise hitzige und scharfe wider den Scharbock dienende Mittel gebraucht als zum Beispiel Löffelkraut, Brunnenkreß, Senf, Meerrettig [*Kren*] und dergleichen, so wird“ dadurch „die Bewegung der scharfen Säfte durch die Gefäße vermehret, welche ohnedem nicht mehr gehörig zusammenhängen, worauf öfters plötzliche und sehr gefährliche Verblutungen erfolgen. Daher haben die gelinden Mittel den Vorzug, welche zugleich aller Fäulniß widerstehen, und die Gefäße stärken. Aus diesem Grunde verdient der Sauerampfer, die Mengelwurz, der Sauerklee vor anderen angepriesen zu werden.“ Erfahrene Ärzte „pflegen zum Löffelkraut allezeit Sauerampfer zu nehmen, ohngeachtet der Scharbock den dritten Grad noch nicht erreicht hat“.

Zum vierten Grad „aber ist selten Hülfe; die Heilungsart ist hier nach Verschiedenheit der Zufälle zu verändern; bisweilen sind die Mittel aus Quecksilber dienlich; als auch die vorgeschriebenen.“

„Wenn die Säfte aus Mangel der Speisen aus dem Pflanzenreiche eine faule stinkende Natur und Beschaffenheit überkommen und angenommen haben“, dann werden die Patienten „blos durch den Genuß der Obstfrüchte und der Brühen von frischem Fleisch und Kohlkräutern auf das glücklichste wiederum hergestellet, wofern“ nicht die „Eingeweide, wegen der faulen Cacochymie [*Verderbnis der Säfte*] bereits stark angegriffen worden sind und Noth gelitten haben“.

Soweit die Therapieanweisungen Gerhard van Swietens.

Einen wesentlichen Beitrag zur Bekämpfung insbesondere des Landskorbuts leisteten die Kartoffeln, die meist zu jeder Zeit im Jahr erreichbar waren. Sie wurden in verschiedenen Zubereitungen in rohem Zustand oder nach Erhitzung verabreicht. Diesbezügliche Erfahrungen sind allerdings schon in der Seefahrt erworben worden. Beweise ihrer Wirksamkeit bringen die Vergleiche des Auftretens von Skorbut in verschiedenen Gefängnissen. Rudolph Krebel [82] zitiert Erfahrungen verschiedener Autoren, dass nämlich „aus den Berichten über Gefängnisse hervorgeht, wie in denjenigen, in welchen Kartoffeln gar nicht oder nur sehr selten verabfolgt werden, der Scorbut eine herrschende Krankheit war, während die Gefängnisse, in denen eine hinlängliche Menge Kartoffeln verabreicht worden [*ist*], ganz davon frei blieben. In mehreren Gefängnissen verschwand der Scorbut gänzlich, so wie nur mehrere Pfunde Kartoffeln der wöchentlichen Diät hinzugefügt worden sind.“ Krebel meint, „aus dem Angeführten erhellt zur Genüge, dass die Kartoffeln in Bezug ihrer Anwendung eine doppelte Bedeutung darbieten: die als alimentöses, und die als therapeutisches Mittel.“

Eine „vortreffliche Wirkung“ zur Heilung des Skorbuts zeigte im Wiener allgemeinen Krankenhause die tägliche Gabe eines „sehr gesättigten Malzaufgusses“ [83]. Eine andere in Österreich und einigen Gegenden Deutschlands bewährte antiskorbutische Maßnahme war der Verzehr von Weißen Rüben, die wie Sauerkraut fermentiert waren [60].

Viele Ärzte verwendeten alle möglichen, uns heute nicht vorstellbaren Mittel zur Therapie des Skorbuts, so z. B. ein k. k. Regimentsarzt die orale Gabe der giftigen Canthariden-Tinktur in Eibisch- oder Graswurzel-Dekokt mit, wie er 1861 schrieb, wunderbarem, rasch und sicher wirkendem Effekt selbst bei „Kranken, welche der Auflösung entgegengingen“ [84].

1862 veröffentlichte Rudolph Krebel eine „Pharmakopoea antiscorbutica“ mit 33 Rezepten zur Behandlung des Skorbuts in Zubereitungen aus verschiedenen Zeiten [8]. Die darin und im Text angegebenen Rezepturen zur oralen Aufnahme betreffen mineralische und vegetabilische Säuren, Blätter, Wurzeln und Rinden verschiedener Pflanzen, Eisen- und Magnesium-Präparate, verschiedene Molken, rohe Erdäpfel und Bierhefen. Zur äußerlichen Behandlung dienen Waschungen und Räucherungen. Die „Behandlung der verschiedenen Complicationen und einiger Symptome“ erfordern „eine besondere Berücksichtigung“. Dazu gehören u. a. Aderlässe, Fiebermittel, Laxantien und Diuretika.

## Die Meinungsvielfalt im 19. Jahrhundert

Jean Bernhard Bornträger (1851–1927) fasste die zu Ende des 19. Jahrhunderts vermuteten Ursachen der Entstehung des Skorbuts so zusammen [41]: alles, „was der Mensch thut und lässt, isst und trinkt, denkt und fühlt, kurzum Alles, was zu dem Erdenbewohner überhaupt in irgendeine Beziehung tritt: Kummer, Langeweile, Furcht, Traurigkeit, Heimweh, Niedergeschlagenheit; Ruhe – Anstrengung, Windstille – Sturm, Kälte – Hitze“, der Wechsel des Klimas, der Jahreszeit und der Witterung, der Art des Dienstes, des Alters, Auftreten von Infektionen. „Vor allem aber wird fehlerhafte Nahrung betont“. Das kann sein: „Gemüsemangel – Gemüsereichthum“, Genuss oder Mangel frischen Fleisches, „Mangel an Eiweiss, an Fetten, an Kohlenhydrate, an Salzen, an Wasser; Alkoholgenuss – Alkoholmangel, Obstmangel – Obstgenuss“, stickstoffreiche oder stickstoffarme Nahrung, Säurenmangel oder -überschuss“, verschiedene Gehalte an Salzen, „Milchmangel, einförmige, schwer verdauliche, reizlose Kost“, unzureichende oder „verdorbene Nahrung (Fleisch, Mehl etc.), wenig oder schlechtes Trinkwasser etc. etc.“.

Auch die Konstitutionslehre wurde bemüht. Der Medizinhistoriker Heinrich Haeser (1811–1884) sah die Entstehung des Skorbuts als Folge einer allgemeinen „skorbutischen Constitution“ [45]. Haesers Schüler Armin Langheinrich [40] erweiterte dies durch die Meinung, Skorbut, Fleckfieber, Englischer Schweiß und Syphilis seien nur als Ausdruck einer und derselben Konstitution anzusehen, nämlich der putriden. Die Bildungsmomente für diese Konstitution suchte er in Witterung, Misswuchs, Teuerung, schädlichen Ortsverhältnissen und in schädlichen Einflüssen der Lebensweise (enge wollene Bekleidung und Schwitzbäder), sowie in anderen physischen, moralischen und politischen Ereignissen. Die allmählich abnehmende Häufigkeit des Skorbuts beruhe auf dem Verschwinden dieser ursächlichen Momente. Überhaupt erklärte er zuletzt, dass der Skorbut vom 15. bis 18. Jahrhundert der einfachste und verbreitetste Reflex gewesen sei, durch den sich jene Konstitution geäußert habe, sodass man ihn selbst als universelle Dyskrasie [*schlechte Mischung der Körpersäfte*] jener Zeiten zu betrachten berechtigt sei und man ihm den wichtigsten Einfluss auf die Pathologie und auf die Erklärung der übrigen Seuchen einräumen müsse.

In der Periode von 1875 bis 1905 herrschte also eine zunehmende Konfusion, wie Kenneth J. Carpenter 1986 rückblickend bemerkte [58]. Im Wesentlichen bestanden aber noch zwei Theorien. Die eine beschuldigte Unregelmäßigkeiten oder Anomalien der Ernährung. Man hielt es für wahrscheinlich, dass die durch Mangel an frischem Gemüse, also durch Mangel an organischen Kaliumsalzen, verursachte Alkalität des Blutes eine Rolle bei der Entstehung des Skorbuts spielt. Aber auch die infektiöse Genese wurde von manchen noch als sicher angesehen [65]. Weiters könnten auch ungesunde Umgebung, Überarbeitung, mentale Depression und Exposition an Kälte und Feuchtigkeit die Entstehung der Krankheit durch Verminderung der Resistenz des Betroffenen fördern. Man dachte aber auch an Vergiftungen durch Ptomaine [*Fäulnisgifte*] aus schlechtem Fleisch und im Falle des kindlichen Skorbuts (Möller-Barlow-Krankheit) unter anderem auch durch bei der Milchsterilisation entstehende giftige Stoffe. Manchen Autoren schien es auch nicht unwahrscheinlich, dass bei der Herstellung von Nahrung spezifische Erreger in diese gelangen oder in ihr entstehen, die dann oral in den Körper aufgenommen werden.

Andererseits ließen die starken Blutungen beim Skorbut auf eine „Alteration des Blutes und der hämorrhagischen Diathese“ schließen, also auf „vermehrte Durchlässigkeit der Kapillaren und feineren Gefäße, bedingt durch unbekannte Veränderungen an denselben infolge der Blutalteration“. Vermutlich zirkuliert „im Blute eine toxische Substanz, die einerseits das Blut, andererseits die Gefäßwand zur Alteration bringt, und ferne schädliche Einflüsse auf innere Organe ausübt“ [43].

Etwas genauer war die Vorstellung von Ludwig Aschoff und Walter Koch auf Grund ihrer Obduktionen von skorbutkranken Soldaten des 1. Weltkriegs [42]. Die wahrscheinliche Ursache des Skorbuts seien dessen hämorrhagische Diathese und „chronische Stoffwechselstörungen als Folge unzweckmäßiger Ernährung, vielleicht in Verbindung mit anderen Einflüssen“, die „am Gefäßsystem des Körpers, wie auch am Stützgewebe eine mangelhafte Bildung oder Veränderung der Kittsubstanzen“ auslösen. Dies bewirkt „wiederum eine größere Durchlässigkeit der Gefäßwände, die zu diathetischen Blutungen^8^ führt, während sich am Stützgewebe selbst, besonders am Knochensystem das Fehlen der Kittsubstanzen in der allgemeinen Osteoporose kenntlich macht“. Wahrscheinlich spielen auch individuelle Dispositionen eine Rolle.

Mit dem Beginn der bakteriologischen Ära in der Mitte des 19. Jahrhunderts hat die Diskussion über die Infektiosität des Skorbuts einen neuen Schwung erhalten. Es wurden Krankheitserreger bei lebenden oder gestorbenen Skorbutkranken im Blut, an der Haut oder an anderen Körperstellen gesucht. Tatsachlich wurden auch ganz verschiedene Bakterien gefunden, die allerdings von den meisten Untersuchern doch nicht für die Erreger des Skorbuts gehalten wurden [41, 42, 85–87]. Andere Untersucher lehnten die infektiöse Genese schon auf Grund ihrer epidemiologischen Erfahrungen ab. Darunter finden sich beispielsweise der englische Arzt James Lind [10], der deutsche Medizinhistoriker August Hirsch [3], der Protomedicus von Ungarn, Franz von Schraud [48] sowie Johann Joseph Čejka (1812–1862) [56] in Böhmen und Rudolph Krebel [8] in Russland. Mit dem Fortschritt der Bakteriologie sprachen sich immer mehr Untersucher gegen eine infektiöse Genese des Skorbuts aus. Schon 1893 erklärte der Hamburger Hafenarzt Karl Reuter (1803–1889), dass Skorbut auf Schiffen mit Bestimmtheit nicht als Infektionskrankheit anzusehen ist und daher sei es verfehlt, „Quarantänemassregeln zu ergreifen oder gar mit Isolierung und Desinfektionsmassregeln sich aufzuhalten“ [88]. Aber noch bis ins 20. Jahrhundert wurde die Frage nach der Infektiosität des Skorbuts erörtert. In epidemiologischen Versuchen an skorbutkranken Meerschweinchen stellte jedoch im Jahr 1912 Viktor Fürst (1870–1961) fest, dass eine „infektiöse Genese für völlig ausgeschlossen“ gelten kann und vermutete einen oder mehrere unbekannte Stoffe in der Nahrung als Ursache der Krankheit [89].

Im Standardwerk der Krankheitserreger, dem „Handbuch der pathogenen Mikroorganismen“, wurden in der 2. und 3. Auflage der Jahre 1903 und 1913 noch Zweifel an einer möglichen Infektionsgenese geäußert, denn „die wenigen sporadischen und unbestätigten Bakterienbefunde klären die Genese keineswegs“ [90]. Während des Ersten Weltkriegs führten Beobachtungen an Kriegsgefangenen zur Vermutung, dass Läuse einen unbekannten Erreger des Skorbuts übertragen könnten [91].

So blieb die Frage nach der Genese des Skorbuts vorerst noch weiterhin nicht geklärt. Es hatte allerdings bereits 1842 der Londoner Professor George Budd (1808–1882) in seinen „Lectures on the disorders resulting from defective nutriment“ [92] festgestellt, dass der Skorbut die wichtigste und am besten bekannte der drei Krankheiten ist, die durch fehlerhafte Ernährung entstehen^9^. Eine Krankheit, der Skorbut, tritt bei längeren Seefahrten auf, aber nur selten an Land und da besonders in Gefängnissen und Asylen, bei Belagerungen und auch verstreut in der armen Bevölkerung von Städten. Die Ursache sei der Ernährungsmangel an saftigem Gemüse oder Früchten oder den daraus zubereiteten Säften. Die prophylaktische Wirkung solcher Nahrungsbestandteile könne nicht auf deren Inhaltsstoffe Albumin, Fibrin und organische Säuren in Verbindung mit anorganischen Basen beruhen, da diese im Blut Skorbutkranker eher in Überfluss enthalten sind. Das wirkende Prinzip, was es auch sei, muss also im Saft vieler Gemüse und Früchte vorkommen.

## Skorbut im Tierversuch

Die experimentelle Erforschung des Skorbuts wurde möglich, nachdem man im Meerschweinchen ein Versuchstier gefunden hatte, bei dem durch bestimmte Ernährung Skorbut erzeugt werden konnte. Es kann also offenbar den „antiskorbutischen Stoff“ nicht wie andere Tiere im Körper selbst produzieren.

Nach den oben angeführten Untersuchungen von Theodor Frølich [71, 72] über die Wirkung der verschiedenen Milcherwärmungen auf die Entstehung von Skorbut führte dessen Zusammenarbeit mit Axel Holst (1860–1931) zu weiteren experimentellen Untersuchungen.

Axel Holst und Theodor Frølich publizierten ab 1907 die Ergebnisse ihrer Versuche mit Meerschweinchen [93–95]. Diese entwickelten die für den menschlichen Skorbut charakteristischen pathologischen Veränderungen, wenn sie einige Zeit jeweils nur mit verschiedenen Getreidearten oder Brot gefüttert worden waren. Ernährung der Tiere mit Weißkohl, Karotten, rohen Kartoffeln, Löwenzahn, Äpfeln oder Zitronensaft verhütete oder heilte die Krankheit. Trocknen oder Kochen des Futters verhinderte oder verminderte diese positive Wirkung.

Eine interessante Beobachtung beschrieb 1912 Valentin Fürst [89]. Das Verfüttern von trockenen Hülsenfrüchten oder trockenen Zerealien an Meerschweinchen hat keine Antiskorbutwirkung. Lässt man diese Samen jedoch auskeimen und verfüttert sie dann, so tritt diese gewünschte Wirkung ein. Eine derartige Empfehlung, nämlich die Abgabe von an Bord gekeimten Sprösslingen des Senfkrauts und der Kresse an Seeleute findet man allerdings bereits im 18. Jahrhundert bei James Lind [10].

Von einigen Untersuchern wurden außer den Meerschweinchen auch Rhesusaffen für Skorbut-Untersuchungen mit Erfolg verwendet [69, 96]. Vielleicht benötigen neben dem Menschen und Rhesusaffen noch weitere Primaten die Aufnahme des Antiskorbutstoffs. Es wurde auch die Frage diskutiert, ob ihn nicht manche Menschen, vielleicht durch eine genetische Mutation, selbst bilden könnten [97].

## Der endliche Erfolg: die Hexuronsäure, das Vitamin C

Die schon eingangs zitierten Erfahrungen auf hoher See, die ja auch zu Lande in vielen Ländern gemacht worden sind, führten immer mehr zur fast allgemein anerkannten Meinung, dass der Verzehr verschiedener Vegetabilien und der Genuss des Saftes von Citrusfrüchten den Skorbut vermeiden und sogar heilen könne.

Trotz der verschiedenen Theorien der Entstehung von Skorbut konzentrierte sich das Interesse auf einen vermuteten antiskorbutischen Stoff in den gegen diese Krankheit wirksamen Nahrungsmitteln. Die Geschichte der Feststellung dieses noch hypothetischen Stoffes schilderten Kenneth J. Carpenter [58] und Leonard G. Wilson [98].

Im Lister-Institut in London untersuchten Arthur Harden (1865–1940) und Sylvester Solomon Zilva (1884–1956) 1918 die Inhaltsstoffe von Zitronensaft. Nach Entfernung von Zitronensäure und anderen organischen Säuren behielt der Saft noch immer seine Wirkung auf Skorbut der Meerschweinchen. Dieser Befund wurde bei Kindern bestätigt. Durch Trocknung ging die antiskorbutische Wirkung verloren. In anderen Ernährungsversuchen an Ratten wurde nach Entfernung des fettlöslichen Faktors A oder des wasserlöslichen Faktors B aus dem Futter durch Zugabe antiskorbutwirksamer Stoffe das Wachstum der Ratten nicht beeinflusst. Waren aber die beiden Faktoren A und B in genügender Menge in der Nahrung enthalten und es wurden Antiskorbutika zugesetzt, so wuchsen die Ratten besser als ohne Letztere. Die Ratten reagierten also auf das Fehlen des antiskorbutischen Faktors durch etwas verzögertes Wachstum, jedoch ohne Symptome des Skorbuts zu entwickeln. Dieser in seiner Art noch unbekannte Stoff wurde dann 1919 von Jack Cecil Drummond (1891–1952) als wasserlöslich erkannt und als Faktor C bezeichnet.

Noch war aber der Antiskorbut-Faktor nicht definiert.

Im April 1932 berichtete Albert Szent-Györgyi (1893–1987), der in Ungarn am Institut für medizinische Chemie der Universität Szeged arbeitete, in einem Brief an den Herausgeber von „Nature“ über das Ergebnis seiner Arbeiten unter der Überschrift „Hexuronic Acid as the Antiscorbutic Factor“ [99]. Die diesbezüglichen Arbeiten hatte Szent-Györgyi bereits in den 1920er-Jahren im biochemischen Laboratorium in Cambridge begonnen. Im Juni 1932 bezeichnete Szent-Györgyi den Stoff, dessen Fehlen Skorbut verursacht, die Hexuronsäure, als Vitamin C [100]. Der Begriff „Vitamin“ war bereits 1912 vom Biochemiker Casimir Funk (1884–1967) im Zuge seiner Untersuchungen von Beriberi geprägt worden. Frank hatte da gefunden, dass diese Krankheit durch Mangel an einem wasserlöslichen Thiamin verursacht wird. Da er damals annahm, dass „Mangelkrankheiten“ allgemein durch Mangel oder völliges Fehlen von bestimmten Aminen verursacht werden, nannte er die für das Leben notwendigen Stoffe „Vitamine“ [*das Thiamin wurde das „Vitamin B1“*]. Albert Szent-Györgyi erhielt 1937 den Nobelpreis für Medizin „in Anerkennung seiner Entdeckung betreffend den biologischen Oxidationsprozess mit besonderer Berücksichtigung des Vitamin C und des Fumarsäurekatalysators“.

Die Hexuronsäure und damit auch das Vitamin C erhielten in der Folge bald den Namen „Askorbinsäure“, gebildet aus dem griechischen „a-“ [*Fehlen von …*] und dem lateinischen „scorbutus“.

Nachdem also die lange gesuchte „Antiskorbutische Substanz“, die Hexuronsäure = Vitamin C, bekannt war, musste ein standardisiertes Maß vereinbart werden. Die ursprünglich definierte Internationale Einheit (IE) war die Wirkung von 100 mg frisch gepressten Zitronensafts. Das wurde bald mit 0,05 mg Askorbinsäure präzisiert. Aktuell wird die Askorbinsäure nur mehr in Milligramm und nicht in IE gemessen.

Die Deutsche Gesellschaft für Ernährung hat gemeinsam mit der Österreichischen und der Schweizerischen Gesellschaft für Ernährung Referenzwerte für die Vitamin-C-Aufnahme 2015 in zweiter Ausgabe herausgegeben, ausführlich besprochen und begründet [101]. Im Jahr 2023 wurden diese Referenzwerte neuerlich bestätigt [102].

Die empfohlene tägliche Zufuhr von Vitamin C beträgt demnach bei Kindern von 0 bis 4 Jahren 20 mg und steigt bis zum Alter von 15 Jahren auf 85 mg. Danach werden für Knaben und Männer Dosen von 105 bis 110 mg und für Mädchen und Frauen von 90 bis 95 mg empfohlen. Schwangere ab dem 4. Monat sollen 105 mg und stillende Frauen 125 mg täglich aufnehmen. Für rauchende Männer und Frauen gelten tägliche Dosen von 155 mg bzw. 135 mg.

In Notzeiten wie während des 2. Weltkriegs wurden Vitamin-C-Tabletten an Soldaten und Schüler abgegeben. Meine Schulkollegen und ich hatten die „Zebion-Tabletten“ täglich mit großer Freude als Ersatz für die damals fehlenden Süßigkeiten genommen.

Bei gewissen „modernen“ Ernährungsweisen, bei denen sich auf Grund besonderer Zusammenstellung der Nahrungsmittel ein chronisch werdender Mangel an Vitamin C einstellt, können trotz ausreichender Energiezufuhr Skorbutsymptome auftreten. Ein bereits vor Jahren in den USA publiziertes Beispiel dafür ist die makrobiotische Zen-Diät [103].

Bei der heute bestehenden leichten Zugänglichkeit zu Vitaminen ist zu hoffen, dass trotz steigender Empfehlungen von Ernährungseigentümlichkeiten der Skorbut weiterhin zu den vergessenen Volkskrankheiten zählt.

## Footnotes

1 *Die eigentliche Kriebelkrankheit ist der Ergotismus, bei dem das Kriebeln (Gefühl des Ameisenlaufens auf der Haut) und die Konvulsionen Hauptsymptome waren* (Flamm [2])

2 *Der Protomedicus war in der Antike der Arzt des Herrschers. In Österreich führte den Titel Protomedicus einerseits der öberste Arzt am Kaiserhof, andererseits der mit der ärztlichen Oberaufsicht in jeder Provinz betraute Arzt. Einige Zeit lang war der Direktor der medizinischen Studien an der Wiener Universität zugleich Protomedicus aller k. k. Erbländer. Die Aufgaben des 1783 geschaffenen Postens eines Protochirurgen waren die Tätigkeiten eines Leibchirurgen des Kaiserhauses sowie als oberster Feldchirurg, Inspektor der Militärspitäler und Direktor der Josephs-Akademie.*

3 Als Walachen wird seit dem 10. Jahrhundert die bodenständige Bevölkerung in der Walachei und Moldau und ab 1862 im vereinigten Königreich Rumänien bezeichnet.

4 Beim Soxhlet-Verfahren wurde jede einzelne Portion der Kindernahrung in eine Soxhlet-Flasche abgefüllt; die danach luftdicht verschlossenen Flaschen wurden gemeinsam 20 min in kochendem Wasser erhitzt und dann bis zum Gebrauch kühl aufbewahrt. Franz von Soxhlet (1848–1926), der „Reformator der Säuglingsernährung“, Wien, München.

5 Kondensmilch: durch Erhitzen auf etwa ein Viertel des ursprünglichen Volumens eingedickte und mit Zucker versetzte (Kuh‑)Milch.

6 Die „Goldene Ader“: Blutgefäße der Hämorrhoiden, deren Blutung oft eine Erleichterung bringt.

7 Theriak: seit dem Altertum bis ins 18. Jh. äußerlich und innerlich anzuwendendes Mittel zur Vorbeugung und Behandlung von Krankheiten; ursprünglich aus 61 Stoffen gemischt, wie z. B. Bezoar (Magenstein von Wiederkäuern), Ambra (fettiges Darmsekret von Pottwalen), Terra sigillata, Korallen, Perubalsam, Hirschhornsalz.

8 Diathese: erblich-konstitutionelle Bereitschaft des Organismus, mit krankhaften Manifestationen an bestimmten Organen oder Organsystemen auf Noxen zu reagieren.

9 Offenbar sind Skorbut, Beriberi und Ergotismus gemeint; es wäre aber möglich, dass noch eine Vierte gemeint war, deren auffälligstes Symptom Diarrhö wäre.

## References

[1] Flamm H. Die Pellagra – vor 250 Jahren im Kaisertum Österreich erstmals beschrieben, wurde sie zu einer lebensbedrohenden Endemie in einigen Provinzen. Wien Klin Wochenschr. 2021;133(Suppl. 1):1–21. 10.1007/s00508-021-01840-z. 33881635 10.1007/s00508-021-01840-z PMC8060190

[2] Flamm H. Der Ergotismus – ein Ackerunkraut aus Mesopotamien wurde in Europa zum noch immer aktuellen Epidemie-Erreger. Wien Med Wochenschr. 2023;173:374–92. 10.1007/s10354-022-00960-z. 36045264 10.1007/s10354-022-00960-z PMC10632199

[3] Hirsch A. Handbuch der historisch-geographischen Pathologie. 2. Aufl. Stuttgart: Ferdinand Enke; 1883. S. 354–96.

[4] Budetzius P. Dissertatio inauguralis medica de Convulsione cereali. Wien: Carl Gerold; 1814. ÖNB Wien: http://data.onb.ac.at/rep/106AA09C. Zugegriffen: 12. Juli 2021.

[5] Fuchs CH. Das heilige Feuer des Mittelalters. Wiss Ann Ges Heilkd. 1834;28:1–81.

[6] Heusinger TO. Studien über den Ergotismus, insbesondere sein Auftreten im neunzehnten Jahrhundert; aus Anlass einer Epidemie in Oberhessen im Winter 1855/56. Marburg: Joh. Aug. Koch; 1856.

[7] Wickersheimer. Ignis sacer – Bedeutungswandel einer Krankheitsbezeichnung. Ciba Symp. 1960;8(1):160–9.

[8] Krebel R. Der Skorbut in geschichtlich-literarischer, pathologischer, prophylactischer und therapeutischer Beziehung. Leipzig: Rudolph Hartmann; 1862. Bayerische StaatsBibliothek, München MDZ.

[9] Forster JR. Geschichte der Entdeckungen und Schiffahrten im Norden. Frankfurt/Oder: Carl Gottlieb Strauß; 1784. S. 273. Bayerische StaatsBibliothek, München MDZ.

[10] Lind J. Treatise on the Scurvy in three parts. 3. Aufl. London: S. Crowder et al.; 1772.

[11] Krantz A. Saxonia. Köln: Johann Soter; 1520. Zit. n. Wierus [14].

[12] Botanologicon CE. Hevs medice. Köln: Johannes Gymnicus; 1534. ÖNB Wien. http://data.onb.ac.at/rep/105CE961. Zugegriffen: 20. Okt. 2022.

[13] Van Wijk N. Frank’s Etymologisch Woordenboek der Nederlandsche Taal. 2. Aufl. Martinus Nijhoff: s’Gravenhage; 1949.

[14] Wierus J. Tractätlein von dem Schurbauch. In Horst [30], pp. 163–96.

[15] Wierus J. De Scorbuto Tractatus. In Ronsseus [19] pp. 215–263; in Sennertus [22], pp. 311–342.

[16] Rolfinck W. Epitome methodi cognoscendi & curandi particulares corporis affectus, secundum ordinem Abubetri Rhazæ ad Regem Mansorem libro nono, Hippocraticis, Paracelsicis et Harveanis principiis illustratæ et recognitæ, Philiatrorum in gratiam adornata & exscripta. Jena: Johann Nisius; 1655. Bayerische StaatsBibliothek, München MD.

[17] Kluge. Etymologisches Wörterbuch der deutschen Sprache. 23. Aufl. Berlin New York: De Gruyter; 1995.

[18] Echthius J. De Scorbuto, vel Scorbutica passione Epitome. In Ronsseus [21] Bl. 26–31 (unter Autor Wierus) bzw. [22] pp. 98–100,201– 214; in Sennertus [23] pp. 299–310 bzw. [24] pp. 181–6.

[19] Biesbrouck M, Goddeeris T, Steeno O. Johann Bachoven von Echt (1515–1576) and his work on Scurvy: An omen of Vesalius’ death. Acta Med Hist Adriat. 2018;16:203–38. 10.31952/amha.16.2.2. Zugegriffen: 1. Nov. 2023. 30488702 10.31952/amha.16.2.2

[20] Pantaleon H. Prosopographiae Herorvm atqve Illvstrivm Virorvm totivs Germaniae, Basel: Officina Nicolai Erylingeri. 1570. https://www.digitale-sammlungen.de/de/view/bsb11055014?page=,1. Zugegriffen: 10. Febr. 2024.

[21] Ronsseus B. De Magnis Hippocratis Lienibus, Pliniiqve Stomacace ac Sceletyrbe, seu vulgò dicto Scorbuto Libellus. 1. Aufl. Antwerpen: Vidua Martini Nutius; 1564. ÖNB Wien: ∗69.M.256(3).

[22] Ronsseus B. De Magnis Hippocratis Lienibvs, Pliniiqve Stomacace ac Sceletyrbe, seu vulgò dicto Scorbuto, Commentariolus. Accessere Eiusdem epistolæ qunqu; eiusdem argumenti. 2. Aufl. Wittenberg: Clemens Schleich; 1585. VD16 R 3015 digidalisiert durch Deutsche Forschungsgemeinschaft.

[23] Sennertus D. De scorbuto tractatus. Cui accesserunt argumenti Tractatus & Epistolæ Balduini Ronssei, Johannis Echthii, Johannis Wieri, Johannis Langii, Salomonis Alberti, Matthaei Martini. 1. Aufl. Wittenberg: Zacharias Schurer; 1624. ÖNB Wien: http://data.onb.ac.at/rep/1088AAA2. Zugegriffen: 27. Juli 2022.

[24] Sennertus D. De scorbuto tractatus. Cui accesserunt argumenti Tractatus & Epistolæ Balduini Ronssei, Johannis Echthii, Johannis Wieri, Johannis Langii, Salomonis Alberti, Matthaei Martini. 2. Aufl. Frankfurt & Wittenberg: Tobias Mevius & Elerd Schumacher; 1654. https://books.google.at/books?id=6__TAQAACAAJ&printsec=frontcover&hl=de&source=gbs_ge_summary_r&cad=0#v=onepage&q&f=false. Zugegriffen: 27. Juli 2022.

[25] Lang J. De Scorbuto Epistolæ duæ. In Ronsseus [21, 22] und in Sennertus [23, 24].

[26] Albertus S. (1.) Schorbuti Historia proposita in publicum. (2.) Theses de Schorbuto medicæ & horum temporum. In: Sennertus [23, 24], pp. 354–573 bzw. pp. 354–540.

[27] Hettenbach E. De Schorbuto, Præside Salomone Alberto. In: Sennertus [23], pp. 541–73.

[28] Martinus M. De Scorbuto Commentario, tribus positionum centuriis comprehensa. In Sennertus [23, 24].

[29] Horst G. Tractatus de Scorbuto seu de magnis Hippocratis lienibus, Pliniique Stomacaceae Scelotyrbe, in illustri Academia Giessena duabus exercitationibus publice in gratiam discentium propositus. Giesen: Nicolaus Hampel; 1609. Bayerische StaatsBibliothek, München MDZ.

[30] Horst G. Büchlein von dem Schorbock / Gemeynem Vatterlandt zum besten Teutsch beschrieben. Mit angehencktem Rath in Pest Zeiten. Auffs newe durchsehen vnd vermehret. Schorbock, pp. 1–133, 148–62; Rath in Pestilentz Zeiten, pp. 133–148; Johañ Weyer, Tractätlein von dem Schurbauch, pp. 163–96. Giesen: Nicolaus Hampel; 1615. Bayerische StaatsBibliothek, München MDZ.

[31] Drawitz J. Bericht und Vnterricht von der Kranckheit des Schmertz-machenden Scharbocks. Woher derselbe entstehet, komme und wie solche Kranckheit zu curiren. Nachdruck d. Aufl. von 1647. Leipzig: Tobias Riese; 1658.

[32] Schwartz WH. De Dolore Lienis, affectu hypochondriaco et scorbuto. In: Rolfinck [16]. Pp. 250–6.

[33] Bierling H. (Præsidio: Rolfinck W.). Disputatio medica inauguralis de Scorbuto. Jena: Lobenstein; 1640. Bayerische StaatsBibliothek, München MDZ.

[34] Blumentrost ML. (Præsidio: Rolfinck W). Disputatio inauguralis de Scorbuto. Jena: Blasius Lobenstein; 1648. Bayerische StaatsBibliothek, München MDZ.

[35] Loëlius JL (Patrocinio: Rolfinck W). ΣΥΖΥΤΗΣΙΣ medica inauguralis de Scorbuto. Jena: Johann Nisius; 1668. Bayerische StaatsBibliothek, München MDZ.

[36] Schulzius G. (Præsidio: Rolfinck W). Disputatio medica sistens ægrum laborantem Febre Tertiana Intermittente Scorbutica. Jena: Johann Werther: 1669. Bayerische StaatsBibliothek, München MDZ.

[37] Van Swieten G. Erläuterungen der Boerhaavischen Lehrsäze von Erkenntniß und Heilung der Krankheiten. 3. Teil, 2. Band. Wien: Johann Paul Krauß; 1755. Pp. 389–465. Bayerische StaatsBibliothek, München MDZ. urn:nbn:de:bvb:12-bsb1036387-3. Zugegriffen: 20. Juli 2022.

[38] Kramer JGH. Medicina castrensis, Das ist: Bewährte Arzney wider die im Feld und Guarnisons unter Soldaten grassirende Krankheiten. 3. Aufl. Wien & Nürnberg: Peter Conrad Monath; 1755. ÖNB Wien. http://data.onb.ac.at/rep/109FADB3. Zugegriffen: 18. Juli 2022.

[39] Rouppe L. De Morbis Navigantium, Liber Unus. Lugdunum Batavorum (Leiden): Theodor Haak; 1764. http://data.onb.ac.at/rep/10B181D5. Zugegriffen: 20. Juli 2023.

[40] Langheinrich A. Scorbuti ratio historica. Dissertatio inauguralis historico-pathologica. Berlin: Nietackian; 1839. Bayerische StaatsBibliothek, München MDZ.

[41] Bornträger J. Skorbut auf Schiffen. Vierteljahresschr Gerichtl Med Öffentl Sanitätswes. 3. Folge. 1893;6:349–76 und Suppl.1:36-55.

[42] Aschoff L, Koch W. Skorbut. Eine pathologisch-anatomische Studie. Jena: Gustav Fischer; 1919.

[43] Sato T, Nambu K. Zur Pathologie und Anatomie des Skurbuts. Virchows Arch. 1908;194:151–82.

[44] Weiß K. Bemerkungen zur Skorbutfrage. Wien Med Wochenschr. 1917;67:257–8.

[45] Haeser H. Lehrbuch der Geschichte der Medicin und der epidemischen Krankheiten. 3. Aufl. Bd. 3. Jena: Gustav Fischer; 1882. S. 389.

[46] Magnus O. Historia de gentibus septentrionalibus. Rom: Giovanni Maria Viotti; 1555. Zitiert v. Hirsch A [3], p. 358, Lind JA [10], p. 300, Langheinrich A [40], p. 22 und anderen Autoren.

[47] van Andel MA. Der Skorbut als niederländische Volkskrankheit. Arch Gesch Med. 1927;19:82–91.

[48] v. Schraud F. Nachrichten vom Scharbock in Ungarn im Jahre 1803, nebst Vorschriften der medicinschen Polizey für nichtansteckende Volkskrankheiten. Wien: Canesianaische Buchhandlung; 1806. Bayerische StaatsBibliothek, München MDZ.

[49] Kramer JGH. Dissertatio epistolica de Scorbuto ad virum Christophorum Jacobum Trew. Norimberga (Nürnberg): Pertr. Conr. Monath; 1737. Univ.-Bibl. Wien (books2ebooks.eu), Sign.I-219247. In deutscher Fassung: Herrn Albrechts von Haller Beyträge zur Beförderung der Geschichte und Heilung der Krankheiten. Aus dessen Sammlung praktischer Streitschriften von D. Lorenz Crell. 3. Band. Berlin und Stettin: Friedrich Nicolai; 1782. pp. 184–222. Universitäts- und Landesbibliothek Sachsen-Anhalt.

[50] Van Swieten G (anonym). Kurze Beschreibung und Heilungsart der Krankheiten, welche am öftesten in dem Feldlager beobachtet werden. Wien, Prag und Triest: Joh. Thomas Trattner; 1758. pp. 144–54. Derselbe Text auch noch in der 3. Auflage (Wien: Johann Thomas Edler von Trattner; 1777. pp. 144–54).

[51] Brambilla JA. Chirurgisch-praktische Abhandlung von der Phlegmone und ihren Ausgängen. 2. Theil, 2. Abtbtheilung, Kapitel III, Von dem Scharbock mit Phlegmone, und dem heißen Brand, der im Jahre 1762 unter den k. k. Soldaten in Schlesien eingerissen hat. pp. 331–354. ÖNB Wien: http://data.onb.ac.at/rep/10A93C41 Zugegriffen: 20. Juli 2022.

[52] Opitz E. Ueber Skorbut. Vierteljahrschr prakt Heilkunde, Prag. 1861;69=NF18/1:108–56.

[53] Chrastina J. Aus dem Wiener Versorgungshause am Alserbach. Oesterr Zeitschr Pract Heilkd. 1859;5:199–200.

[54] Kirchenberger S. Die Scorbutepidemie in der Prager Garnison im Jahre1873. Vierteljahrschr prakt Heilkunde, Prag. 1874;3(Orig.-Aufs 123):33–52. ÖNB Wien. https://anno.onb.ac.at/ogi-content/anno-plus?aid=vph&datum=1874&page=689&size=45. Zugegriffen: 28. Juni 2023.

[55] Beer L., Über die in öffentlichen Straf- und Correctionshäusern vorkommende scorbutische Cachexie. Med Jahrb k.k. österr Staates. Wien 1844. 49. & 50. Band = NF 40. & 41. Band. 294–299.

[56] Čejka (JJ). Scorbutepidemie im k. k. Provincialstrafhause zu Prag im Monate Mai und Juni 1843. Vierteljahresschr Heilkunde, Prag. 1844;1/2. Quartal:7–37.

[57] Paul. Einiges zur Pathologie des Skorbuts in Gefängnissen. 33. Jahresbericht Schlesische Gesellsch für vaterländ Kultur. Breslau: Graß, Barth u. Comp.; 1855. p. 133–7. https://www.biodiversitylibrary.org/bibliography/50438 Zugegriffen: 3. Juli 2022.

[58] Carpenter KJ. The history of scurvy and vitamin C. Cambridge: Cambridge University Press; 1986.

[59] Glisson F. A Treatise of the Rickets being a Disease Common to Children. London: Peter Cole; 1651. Zit n Carpenter [58].

[60] de Mertans C. Observations on the scurvy. Observations sur la Scorbut. Phil Trans Roy Soc Lond. 1778;68/II:661–80.

[61] Frank JP. Discursus academicus de rachitide acuta, et adultorum. Delectus opusculorum medicorum antehac in Germaniae diversis academiis editorum. 1788;5:304–18.

[62] Moeller (JOL). Zwei Fälle von acuter Rachitis. Königsberger Med Jahrb. 1862;3:135–49.

[63] Cheadle WB. Three cases of scurvy supervening on rickets in young children. Lancet. 1878;ii:685–7.

[64] Barlow T. On cases described as ‘acute rickets’ which are probably a combination of scurvy and rickets, the scurvy being an essential, and the rickets a variable, element. Med Chir Trans. 1883;66:159–220. Reprint: https://www.ncbi.nlm.nih.gov/pmc/articles/PMC1975441/pdf/archdisch01491-0018.pdf. Zugegriffen: 10.03.2023. 20896608 10.1177/095952878306600112 PMC2121454

[65] Berthenson L. Zur Statistik und Aetiologie das Scorbuts. Die Scorbutepidemie von 1889 nach Beobachtungen im St. Petersburger Nikolai-Militärhospital. Dtsch Arch Klin Med. 1892;49:127–55, 323–47.

[66] Salmenperā L. Vitamin C nutrition during prolonged lactation: optimal in infants while marginal in some mothers. Am J Clin Nutr. 1984;40:1050–6. https://academic.oup.com/ajcn/article-abstract/40/5/1050/4691421?redirectedFrom=fulltext. Zugegriffen: 27.07.2022. 6496385 10.1093/ajcn/40.5.1050

[67] Neumann H. Bemerkungen zur Barlow’schen Krankheit. Dtsch Med Wochenschr. 1902;28:628–30,647–9.

[68] Hess AF. Infantile scurvy. A study of its pathogenesis. Am J Dis Child. 1917;14:337–53.

[69] Hart K. Über die experimentelle Erzeugung der Möller-Barlowschen Krankheit und ihre endgültige Identifizierung mit dem klassischen Skorbut. Virchows Arch. 1912;208:367–96.

[70] v. Stark. Ueber Scorbutus infantum. 73. Versamm.Dtsch Naturforsch Aerzte Hamburg, 22.– 28.09.1901. Ref. Wien Med Wochenschr. 1902;52:765.

[71] Frölich T. Ueber die Ursachen von Skorbut. Norsk Magaz for Lägevid 1910:252, ref. Wien Med Wochenschr. 1911;61:405–6.

[72] Frölich T. Experimentelle Untersuchungen über den infantilen Skorbut. Z Hyg. 1912;72:155–82.

[73] Bartholomew M. James Lind and scurvy: a revaluation. J Marit Res. 2002;4(2022):1–14. 10.1080/2153369.2002.9668317. Zugegriffen: 26. Juni 2022. 10.1080/21533369.2002.9668317 20355298

[74] Altmann CN. Dissertatio Inavgvralis Medico-chemica Analysim Plantarvm Antiscorbvticarvm, et Tentamina: Num in iis Sal Volatililis Alcalinus præexistat? Wien: Wwe. G. L. Schulz; 1766. ÖNB Wien: http://data.onb.ac.at/rep/10AFAA4FF. Zugegriffen: 27. Juli 2022.

[75] Agricola J. Medicinae Herbariae Libri duo. Basel: Bartholom. Westhemerus; 1539. ÖNB Wien. http://data.onb.ac.at/rep/1084B120. Zugegriffen: 20. Okt. 2022.

[76] Vietz FB. Abbildungen aller medicinisch-ökonomisch-technischen Gewächse, mit der Beschreibung ihres Nutzens und Gebrauches. 9. Band. Wien: Schämblischer Bücherverlag; 1818. ÖNB Wien. http://data.onb.ac.at/rep/10501865. Zugegriffen: 20. Okt. 2022.

[77] Moellenbrock VA. Cochlearia curiosa, cum figuris et indice locupletissimo. Leipzig: C. Ulmann für J. Gross; 1674 und 1746.

[78] Sherley T. Cochlearia curiosa: or the curiosities of scurvygrass. London: Griffin for William Cademan; 1676.

[79] Reichenbach L. Icones Florae Germanicae et Helveticae. 3. Band. Leipzig: Friderich Hofmeister; 1838–1839. ÖNB Wien. http://data.onb.ac.at/rep/10A630EC. Zugegriffen: 20. Okt. 2022.

[80] Mayer M. Inaugural-Dissertation: Verständnis und Darstellung des Skorbuts im 17. Jahrhundert. Würzburg: Universität; 2012.

[81] Nocht B. Ueber Citronensaft als Vorbeugungsmittel gegen Skorbut an Bord. Arch Schiffs Tropen Hyg. 1899;3:109–11.

[82] Krebel R. Ueber den Nutzen der Kartoffeln zur Beseitigung von Scorbut. Med Z Russ. 1856;13:36–8. Bayerische StaatsBibliothek, München MDZ.

[83] Schraud F. Abhandlung von der Verbindung der Lustseuche mit dem Scharbocke und desselben Heilungsart. Wien: Joseph Edler von Kurzbeck; 1791. http://digital.slub-dresden.de/id416034888.69. Zugegriffen: 21. Juli 2023.

[84] Derblich. Zur Aetiologie und Therapie des Skorbuts. Wien Med Wochenschr. 1861;11:811-4,825-7.

[85] Babes V. Ueber einen die Gingivitis und Hämorrhagien verursachenden Bacillus bei Skorbut. Dtsch Med Wochenschr. 1893;19:1035–7.

[86] Kamen L. Zur Aetiologie des Skorbuts. Intern Klin Rundschau. 1888;2:972–3.

[87] Hušša F. Beitrag zur Kenntnis des Skorbuts. Wien Med Wochenschr. 1912;62:2199–2203, 2252–5.

[88] Reuter K. Ueber Skorbut und Beriberi auf Kauffahrteischiffen und deren Verhütung und Behandlung vom Standpunkte der öffentlichen Gesundheitspflege. Vierteljahresschr gerichtl Med u öffentl Sanitätswesen. 3. Folge. 1893;31:Suppl. 101–48.

[89] Fürst V. Weitere Beiträge zur Ätiologie des experimentellen Skorbuts des Meerschweinchens. Z Hyg. 1912;72:121–54.

[90] Handbuch pathogener Mikroorganismen (Kolle W, Wassermann A, Hsg.), 2. Aufl, 3. Band, p. 903; 3. Aufl, 7. Band, p. 930. Jena: Gustav Fischer; 1903 bzw. 1913.

[91] Much H, Baumbach K. Skorbut. Munch Med Wochenschr. 1917;64: Feldärztliche Beilage Nr. 26: 406.

[92] Budd G. Disorders resulting from defective nutriment. London Med Gaz 1842;2:632–6. Reprint. https://wellcomecollection.org/works/b8epyvag. Zugegriffen: 10. März 2023.

[93] Holst A, Frölich T. Experimental studies relating to ship beri-beri and scurvy . II. On the ecology of scurvy. J Hyg. 1907;7:634–71. 20474337 10.1017/s0022172400033623 PMC2236195

[94] Holst A, Frölich T. Ueber die Ursachen von Skorbut. Norsk Magaz for Lägevid. 1910:209, ref. Wien Med Wochenschr. 1911;61:406.

[95] Holst A, Frölich T. Über experimentellen Skorbut. Ein Beitrag zur Lehre von dem Einfluss einer einseitigen Nahrung. Z Hyg. 1912;72:1–120.

[96] Harden A, Zilva SS. Experimental scurvy in monkeys. J Path Bact. 1919;22:246–51.

[97] Cummings M. Can some people sythesize ascorbic acid? Am J Clin Nutr. 1981;34:297–8. https://academic.oup.com/ajcn/article/34/2/297/4692961?login=true Zugegriffen: 26.01.2023.. 7211730 10.1093/ajcn/34.2.297

[98] Wilson LG. The clinical definition of scurvy and the discovery of vitamin C. J Hist Med Allied Sci. 1975;30:40–60. 1094060 10.1093/jhmas/xxx.1.40

[99] Svirbely JL, Szent-Györgyi A. Hexuronic acid as the antiscorbutic factor. Nature. 1932;129:576.

[100] Szent-Gyorgyi A. Vitamin C. Nature. 1932;129:943. 10.1042/bj0260865 PMC1260981 16744896

[101] Deutsche Gesellschaft für Ernährung.. New Reference Values for Vitamin C Intake. Ann Nutr Metab. 2015;67:13–20. 26227083 10.1159/000434757

[102] Deutsche Gesellschaft für Ernährung.. Vitamin C – Referenzwerte 2023. https://www.dge.de/wissenschaft/referenzwerte/vitamin-c/. Zugegriffen: 26. Febr. 2023.

[103] Sherlock P, Rothschild EO. Scurvy produced by Zen macrobiotic diet. J Am Med Ass. 1967;199:794–8. 6071292

[104] Hirschsprung H. Die Moeller’sche Krankheit. (Synon.: „Acute Rachitis“, Scorbut bei Kindern, Barlow’sche Krankheit, Cheadle-Barlow’sche Krankheit etc.). Jahrb Kinderheilkd. 1896;NF41:1–43.

